# A supranational solidaristic space? Comparative appraisal of determinants of individual support for European solidarity in the COVID-19 era

**DOI:** 10.1057/s41295-023-00345-5

**Published:** 2023-04-07

**Authors:** Luís Russo

**Affiliations:** Department of Political and Social Sciences, European University Institute, Badia Fiesolana - Via Dei Roccettini 9, 50014 San Domenico di Fiesole, FI Italy

**Keywords:** European Union, European solidarity, COVID-19, Public opinion, Survey research

## Abstract

COVID-19 created profound shockwaves across the Union, pushing supranational crisis policymaking to the forefront of European politics and fostering an unprecedented expansion in fiscal solidarity with which to support the economic recovery ahead. This development lends pertinence to a contemporary reappraisal of the main determinants underlying individual support for European solidarity and its implications to the consolidation of a political basis for a supranational solidaristic space. Using an original large-N survey dataset and employing a fixed-effects linear regression analysis, this paper empirically reviews ideal-type theoretical predictions for individual support for European solidarity by conducting a comparative assessment of their correlates' explanatory power in the new pandemic context. First, I contend individuals reason in supranational terms as key political attitudes driving individual support for cross-border solidarity are informed directly at the supranational level, consubstantiating the claim that European redistribution operates as a distinct legitimate space for solidarity in its own right. Second, I argue that utilitarian motivations linked to expectations of material amelioration are better predictors of support for solidarity than cultural explanations emphasising national identity or attitudes towards immigration. Third, I suggest that preferences concerning European solidarity are better captured by political divides over economic redistribution rather than over cultural concerns, but only among more cosmopolitan-oriented individuals. In any event, cultural factors are still relevant predictors of support for solidarity, particularly among nationalists. The final section interprets these findings by discussing how the correspondence between public expectations and institutional supply of supranational redistributive instruments to respond to the pandemic may contribute to strenghten political support for European solidarity and the EU polity itself.

**Supplementary Information:**

The online version contains supplementary material available at 10.1057/s41295-023-00345-5.

## Introduction

As highlighted in this issue, COVID-19 did not menace the foundations of public support for the EU polity; previous empirical studies suggest that support for EU membership and supranational solidarity remained remarkably stable despite costly and experimental cross-national redistribution instruments (Genschel et al 2021). What lessons can we draw for the *bonding* dimension of the EU polity (Ferrera 2005)? As European economies grinded to a halt, the economic expression of the pandemic crisis gained widespread salience; indeed, this article finds expectations of economic amelioration to be the main motivation for supporting EU solidarity in this period. In turn, the EU championed crisis policymaking addressing economic recovery and resilience, particularly with Next Generation EU. At a time when EU solidarity was exceptionally salient and well received, the match between public demand for and institutional supply of *material* solidarity might explain why such a ‘fragile experimental polity’ (Oana et al 2023) weathered the crisis much better than expected; instead of unleashing disintegration and populist contestation, the delivery of strong EU-level emergency policies that suited public expectations may have rather contributed to the consolidation a political basis for a *supranational* solidaric space—itself an expression of public *bonding* to a supranational political community.

The pandemic context in 2020 and 2021 is relevant for several reasons: first, its epidemiologic and economic shock generated deep-seated social, health and economic consequences, pushing the salience of supranational solidarity and the EU’s crisis managing role to forefront of European politics. Second, the exogenous nature of the shock may have contributed to the mustering of political capital for supranational solidarity among European publics, drawing from Genschel and Hemerijck’s (2018) expectation that exogenous crises elicit stronger support for solidarity than endogenous imbalances, perceived to be less deserving of transnational relief. Third, this reserve of goodwill for EU solidarity was contemporary to a period of EU policy experimentalism, whereby the EU adopted unprecedented solidaristic instruments to mitigate the fallout of COVID-19, particularly in the (beforehand unfathomable) realm of fiscal solidarity.

This positive context for solidarity contrasts with an *ex ante* state of affairs where solidarity has been in high demand but low supply (*Ibid.*). On the one hand, the increasing interdependence between EU member states and the scope of recent crises have generated significant cross-border pressures and spill overs, clearly outlining the case for a *EU-wide* solidaristic net with which to mitigate these effects. On the other hand, European solidarity is costly, requires intertemporal trade-offs, may face risks of moral hazard and is politically far from unchallenged in a Union characterised by an ambiguous polity (Mair 2007), the absence of a demarcated demos (De Wilde and Trenz 2012) and where polity attachment is considered ‘inherently fragile’ (Hobolt and de Vries 2016). In short, the relatively cautious, discrepant and conditional nature of support for cross-border redistribution has generated a tension that may have hindered more ambitious plans for European solidarity in the past (particularly during the Great Recession); with the pandemic, the EU ably seized the public’s exceptional willingness towards cross-national solidarity as an enabler of a more ambitious redistributive policymaking.

Public opinion plays a fundamental role in the design of European solidarity and in the sustainability of the EU polity itself. Taking into consideration the increasing politicisation of the EU in domestic political arenas (Hooghe and Marks 2009), intergovernmental negotiations concerning the extent and configuration of solidarity are chiefly informed by domestic electoral pressures, giving national voters a non-negligeable role in conditioning the outcome of solidarity supply in the EU. As such, appraisals of public opinion underlying EU solidarity are key to predict its shape, nature and durability, and the political compromises that are required for it to materialise. I argue this research endeavour is ever more pertinent in this period of above-mentioned goodwill towards European solidarity and expansionist solidarity policymaking; should motivations behind support for solidarity be tied to expectations of *material* amelioration and provided that the economic recovery packages are successfully implemented, I harbour the expectation this can positively cement political capital for EU solidarity in the long run, eliciting the need for this analysis at this time.

There is a significant number of important contributions on individual support for European solidarity. Many empirical studies are set against the background of the euro crisis, focussing on fiscal solidarity particularly within the context of this period (e.g. Beaudonnet 2014; Ciornei 2014; Kuhn and Stoeckel 2014; Daniele and Geys 2012, 2015; Kuhn et al 2018; Verhaegen 2018), but scholarly attention to drivers of support for EU solidarity has faded somewhat since then. Few studies were published during COVID-19—Bauhr and Charron (2021) being a notable exception, although with a different design and a more limited geographical scope. Some authors test correlates of solidarity without controlling for self-interest correlates (e.g. Ciornei 2014) or using them only as moderators of another, more central explanation (e.g. Verhaegen 2018; Kleider and Stoeckel 2018; Vasilopoulou and Talving 2020); others model utilitarian correlates as contextual controls, such as living in a creditor/debtor state (Lengfeld et al 2015; Daniele and Geys 2015; Verhaegen 2018) or, more frequently, as individual-level attributes such as social class (Kuhn and Stoeckel 2014; Kleider and Stoeckel 2018), income and occupation (Verhaegen 2018; Kuhn et al 2018; Vasilopoulou and Talving 2020; Bauhr and Charron 2021) or perceptions on own financial situation (Beaudonnet 2014; Ciornei 2014; Verhaegen 2018). This may be suboptimal as evidence suggests sociotropic economic perceptions are more influential factors for political preferences in general (Stegmaier and Lewis-Beck 2013) and for EU solidarity in particular (Bauhr and Charron 2021) than egotropic economic perceptions; also, individual socioeconomic status and perceptions concerning national economic performance are, in reality, *approximations* to the concept of perceived self-interest in European redistribution, while not measuring it directly.

Based on a novel large-N survey dataset on attitudes towards European solidarity, this paper assesses which main determinants operate behind individual support for European solidarity, and by extension which theoretical postulate is better able to encapsulate citizens’ motivations to support or oppose supranational redistribution. It departs from previous insights by analysing utilitarian correlates not as controls or moderators but as key independent variables alongside other explanations such as cultural affinity and political ideology; and, chiefly, by using an indicator that *directly* measures *sociotropic* expectations of net-benefit/loss from EU solidarity to one’s own country, thus making it potentially better suited to estimate the link between national self-interest and support for solidarity. It aims to innovate also by empirically illustrating whether a distinct supranational solidaristic space is recognised among European publics and by borrowing on EU integration and cleavage theory studies to establish a revisited theoretical paradigm and empirical framework which clearly captures the main motivations underlying support for EU solidarity, as an expression of an engagement with the EU polity.

This paper addresses three research agendas: first, it analyses whether support for solidarity is informed directly at the supranational level or whether it is a mere extrapolation of attitudes towards domestic political institutions. Second, it measures the comparative strength of materialist and cultural explanations for support for solidarity by assessing which correlates (advanced by both theoretical underpinnings) better predict this support. Finally, it will borrow insights from cleavage theory in order to disentangle whether EU solidarity support follows a traditional material left–right divide or, alternatively, a demarcation/integration transnational divide. The theoretical overview section will develop a typology of extant theoretical contributions to be empirically assessed; the design section will describe the methodology; the following section discusses results; and the papers concludes with a brief discussion on the implication of these findings for the future of European solidarity and the EU polity.

## Theoretical overview

### Concept of European solidarity

European solidarity is conceptualised in this paper as *fiscal transfers* between *member states*, so-called *member state solidarity* (Fernandes and Rubio 2012), as opposed to *transnational solidarity* highlighting cross-border welfare provision to EU citizens (Sangiovanni 2013). Lengfeld et al (2015) apply Weber’s (1968, pp. 24–25) instrument- and value-oriented political legitimacy typology to support for EU solidarity, theorising that individuals broadly subscribe to it either due to self-interest or because of a belief in its moral appropriateness and communal duty, described, respectively, as *zweckrational* and *wertrational* causes for solidarity. I take heed from this dichotomy to structure the theoretical discussion in this paper. Additionally, given the relative scarcity of theoretical accounts on individual determinants of support for EU solidarity, I will draw on the literature on individual attitudes towards EU integration due to the (at least theoretical) adjacency between the two—support or opposition to EU solidarity can be a manifestation of the desire or refusal to belong to the European polity, something that has been suggested by some empirical evidence (Kuhn et al 2018; Kleider and Stoeckel 2018; Baute et al 2019).

### Levels of European solidarity

The question of whether EU citizens see their national community as the sole legitimate level for redistribution or whether they also contemplate a *supranational* solidaric space is a very relevant one. Some authors posit the so-called *proxy theory* (Armingeon and Ceka 2014) whereby citizens’ attitudes towards the EU (and in this case, EU solidarity) would be an extrapolation of attitudes towards their respective national institutions. Others posit that political support for the EU and European solidarity derives from political attitudes concerning the supranational polity directly. In that case, views on EU membership and trust in EU institutions are expected to greatly matter to individual support for solidarity—at least to a greater extent than trust in national institutions. This has not been yet empirically probed in a comparative fashion, as there are no assessments regarding the level at which political attitudes correlate more strongly with support for European solidarity.

Genschel et al (2021) found that, in 2021, a majority of Europeans supported European solidarity. Additionally, 67% thought their country should provide major help to other countries in case of a pandemic and 68% believe that effort should be part of a joint effort managed by the EU. This supports the expectation that, either by material interest or cultural affinity, Europeans generally supported redistribution at the *supranational* level during the pandemic. Additionally, findings that Europeans are more willing to provide help to EU rather than non-EU countries suggests that European integration has established an inner group for supranational solidarity (Lengfeld and Kroh 2016) and that ‘a strong mobilisation against European solidarity measures is unlikely’ (Lengfeld et al 2015, p. 21). During COVID-19, it is likely that the expansion of the EU’s role the economic recovery may have pushed its stimulus package to the centre of domestic debates, thereby increasing the salience of a solidarity framework led by the EU. Consequently, it is expected that:

#### H1

Support for EU solidarity is more strongly correlated with political attitudes concerning supranational institutions than national governments.

### Determinants of European solidarity

Extant scholarship on support for solidarity is informed by three main theoretical postulates, as detailed in Table 1. These theoretical underpinnings can be grouped into two dimensions: the first deals with the community level which more strongly informs preferences for solidaric protection (national or supranational); and the second concerns the substantive nature of the motivations underlying support for solidarity (material or cultural).

**Table 1:** Ideal-type typology for individual support for solidarity

	Nature of preferences	
	Material	Cultural
*Solidarity preference for*		
National community	Utilitarian	Protectionist / Cosmopolitan
	Benefit/loss calculus from European solidarity to the *national* community (sociotropic utilitarianism) Relevance of economic left–right divide (left supporting, right opposing)	Identity (stronger national identity associated with nation state as sole legitimate solidarity provider/ stronger multiple/European idenitities associated with stronger support for supranational solidarity) Attitudes towards migration (supporters of open borders and multiculturalism more prone to support EU solidarity/ less multicultural individuals prone to oppose it) Relevance of cultural integration/demarcation divide
Supranational community	Prioritisation of a social agenda for the EU Benefit/loss calculus from European solidarity to the *supranational* community Relevance of economic left–right divide	

Within material-based accounts, utilitarian explanations are operationalised here at the sociotropic level, stressing  economic cost-benefit calculus (Downs, 1957) concerning perceived gains arising from European  redistribution into the national level (e.g. Daniele and Geys, 2015; Rodríguez-Pose and Dijkstra, 2021), while social explanations highlight social equity and redistributive fairness underpinning a supranational social agenda for European solidarity aiming at mitigating social imbalances within the Union (Corneo and Grüner, 2002; Fernandes and Rubio, 2012; Vandenbroucke, 2013). Cultural-based accounts emphasise the role of (exclusive) national identity and perceived cultural threat in shaping the notion of the nation state as the sole legitimate provider of solidarity for the national community and, by contrast, how cosmopolitan attitudes accentuating individual attachment to multiple identities may act as an enabler for cross-national solidarity support (e.g. McLaren, 2002, Kuhn et al, 2018, Bauhr and Charron 2021). These theoretical accounts and suggested correlates are further detailed in online Appendix I. While it is expected that most are relevant explanations to some degree, assessing *how much* they account for total variation in support for solidarity by comparatively measuring their predictive power is a novel and, I believe, a worthwhile research operation, as it enables to empirically ascertain which theory is better equipped to explain Europeans’ support for solidarity during COVID-19.

The central piece of European solidarity efforts in the (post-)pandemic period is the Next Generation EU programme. Given the distinctly economic nature of the crisis and the EU’s response to it, it could reasonably be expected that support for solidarity is strongly connected to domestic redistribution gains with which to launch the economic recovery ahead, stating the case for utilitarian determinants (net-benefit perceptions) to be stronger correlates of support for solidarity than social (preference for a social European model) and cultural (national identity and immigration insecurity) ones. This raises the expectation that:

#### H2

Utilitarian determinants are more strongly correlated to support for European solidarity than cultural or social determinants.

### Divides on European solidarity

Drawing on the use of cleavage theory underpinning attitudes towards the EU (Hooghe et al 2002; Hooghe and Marks 2017), one could expect to find a cleavage approach useful in understanding divisions concerning support for EU solidarity. The *cultural* transnational divide stresses the role of national identity, economic insecurity and immigration under what Kriesi et al (2006; 2012) call the demarcation/integration divide, conceptualised as the conflict between the winners of globalisation (supporting transnational integration and open borders) against those who desire to protect national culture, oppose international trade and resist immigration due to their lower levels of skill and economic perspectives. Orthogonally to that divide, some suggest EU solidarity follows a traditional economic left–right divide (e.g. Bechtel et al 2014; Daniele and Geys 2015; Kleider and Stoeckel 2018) where support for solidarity gravitates around issues of economic redistribution and inequality, with the left typically in favour of more redistribution to increase equity and social fairness (Fong 2001), and the right typically opposing demand-side policy and state intervention in the economy (Hooghe and Marks 1999). In theory, the cultural divide should correspond to ‘protective/cosmopolitan’ explanations, whereas the economic left–right divide would operate within materialistic ‘utilitarian’ or ‘social’ explanations. For more detail, please consult the theoretical map outlined in online Appendix I.

To the best of this author’s knowledge, an operationalisation of competing cleavages underpinning support for EU solidarity has not yet been conducted in previous academic appraisals of the subject matter, consubstantiating an innovative element of this paper’s argument. Consistent with the overriding argument that material expectations highlighting net-benefit/loss are better able to explain support for solidarity than cultural determinants, it follows that a traditional left–right divide (measured here by left–right self-placement), more akin to economic redistribution concerns, is expected to better predict support for solidarity than views on immigration and national identity, associated with a cultural demarcation/integration divide.

#### H3

Support for European solidarity is better predicted by an economic left–right divide than by a cultural integration/demarcation divide.

## Design

### Data and analysis

The analysis in paper will draw on a novel and original dataset (Hemerijck et al 2021) extracted from a survey developed for the ‘Solidarity in Europe’ research project at the European University Institute (EUI), implemented by YouGov in cooperation with the EUI. Our survey is, to the best of our knowledge, the largest survey dedicated to individual attitudes towards European solidarity, collecting data from around 23,000 respondents each year, across 13 member states: Denmark, Finland, Sweden, the Netherlands, Spain, Germany, Lithuania, Hungary, Romania, France, Poland, Greece and Italy. Missing and ‘don’t know’ responses were removed from the analysis, setting the final sample size at 18.062 respondents who chose a non-ambivalent option on all indicators. The dataset used in this paper was extracted from the survey’s third and fourth wave, fielded online from 17 to 29 April 2020 and from the 12 to 27 April 2021, respectively. Through a controlled panel sampling mechanism implemented by YouGov, the sample is representative of the population in each country concerning age, gender, social class, region, level of education, vote in the previous election and level of political interest.

I will employ a fixed-effects linear regression, clustered by country, to inspect the how variables identified within each ideal-type hold in accounting for variation in support for European solidarity. As this paper has the ambition of establishing inferences for the European population, the level of analysis will be set at the supranational level. This occurs for three reasons: first, it can be theoretically expected that variation in support for solidarity is inspired by individual subjective dispositions rather than a result of domestic economic, social and political conditions (Daniele and Geys 2015); second, the context under which this paper is developed (COVID-19 as a booster of salience and political capital for solidarity) is expected to affect all countries similarly due to the cross-national EU-wide effects of the pandemic shock; and third, due to the finding that, in the dataset in point, 94% of variation of solidarity support occurs across individuals rather than across countries. Given the theoretical relevance of measuring the relative magnitudes of determinants for solidarity support, they will be included in a single model to control for mediating and confounding effects. The mean variance inflation factor is 2.32, confirming that no multicollinearity is observed in the model, below the recommended cut-off value of 10 (Meuleman et al 2015).

Because it can be theoretically assumed that all ideal-type correlates can explain support for solidarity to a certain extent, their effect sizes will be standardised and comparatively assessed, allowing to identify which correlates account for the highest share of variation in support for EU solidarity, even if originally expressed in different scales. By empirically testing the extant theories and the magnitude to which they can explain observed solidarity, we argue we can ascertain which theory is better equipped to predict public support for European solidarity. These results and their implications be discussed final section of this paper.

### Variables

All variables will be extracted from the EUI-YouGov ‘Solidarity in Europe’ dataset. The dependent variable used in the model is an indicator measuring preferences towards redistribution within the EU, composed of a 11-point Likert scale where 0 means the respondent thinks resources should be spent only on her own country and people, while 10 means supporting spending resources equally on all countries and all people in the European Union. In line with our theoretical assumption, the dependent variable aims to capture support for European solidarity by measuring individual preferences regarding redistribution of fiscal resources at the supranational level. To account for variation in this variable, a set of theoretically-derived independent variables will be employed.

For H1, to test whether support for solidarity is informed by national or the supranational political attitudes, the model will include indicators on *attitudes towards EU membership* (measured by whether respondents would vote to leave or remain in the EU in case of a referendum on EU membership), *trust in EU institutions* and *trust in national governments* (to what extent respondents trust the EU or national governments to make things better, respectively). The two latter variables are indexes built on respondents’ answers to that question over 11 different policy areas (*vide* online Appendix IV). The *trust in government* variable will control for a potential mediating association between trust in EU and support for solidarity deriving from an extrapolation to the EU level of attitudes towards domestic governments (Armingeon and Ceka 2014). These variables will thus allow to test H1 on whether political attitudes operating behind support for solidarity are directed at the supranational level or are contingent on the domestic level.

H2 inquires whether the main correlates of European solidarity correspond to those forwarded by material or cultural theoretical accounts. Utilitarian determinants will be operationalised by a binary variable gauging *net-benefit* expectations, i.e. whether respondents think their country is a net-winner (receives more than contributes) or net-loser (contributes more than receives) from a hypothetical EU-wide crisis solidarity fund. Cultural determinants are measured by two variables: *identity* (only national/national and European/European and national, only European) and feelings of insecurity regarding immigration (very secure/fairly secure/fairly insecure/very insecure). *Immigration insecurity* has been used in numerous studies as a relevant indicator for culture-based explanations for attitudes towards European integration, namely those emphasising a perceived cultural threat, opposition to multiculturalism and defence of national culture (e.g. Carey 2002; de Vreese and Boomgaarden 2005; Hobolt and Brouard 2011; Hooghe and Marks 2017). A variable measuring preference for a ‘Market’ (stressing economic integration, market competition and fiscal discipline), ‘Protective’ (stressing the defence of the European way of life and welfare against internal and external threats) or ‘Global’ (stressing European leadership on climate, human rights and global peace) *models for Europe* will test the social argument by measuring the correlation between support for solidarity and preferences for a ‘Protective’ European model (as a proxy for support for a EU social agenda).

**Fig. 1:**
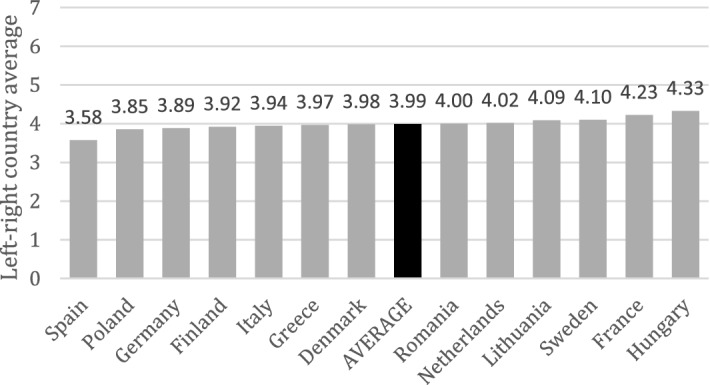
Left–right self-placement country means

H3 assesses whether the cleavage concerning support for European solidarity follows a cultural integration/demarcation or an economic left–right divide. Some authors link the emergence of the former to strong and exclusionary national identity predispositions opposing supranational integration and cultural intermixing, orthogonally to left–right political ideology (Kriesi et al 2012). The relevance of this divide will be appraised by the relative explanatory power of *identity* and *immigration insecurity* in the model. In contrast, scholars supporting the latter (e.g. Kleider and Stoeckel 2018) find that economic left–right orientations are key predictors of public support for supranational solidarity. A Likert 7-point *left–right self-placement* indicator (from left = 1 to right = 7) will explore traditional economic left–right. A caveat to this approach has to do with a potential lack of translatability of ‘left’ and ‘right’ labels across countries—however, the use of a left–right scale is common practice in comparative political analyses in Europe, and the very limited dispersion of the left–right country means (Fig. 1) and very similar standard deviations across countries (online Appendix VI) lends some confidence to the use of these labels for comparative purposes in the surveyed countries.

A set of controls used abundantly in the literature on support for solidarity will be used (*age*, *subjective economic position*, *gender*). *Age* is grouped into five age groups; *subjective economic position* measures one’s reported economic situation in comparison with other same-aged nationals, and *gender* is divided into female and male. Finally, the model will also control for yearly variation between 2020 and 2021 by adding a binary *year* variable.

**Fig. 2:**
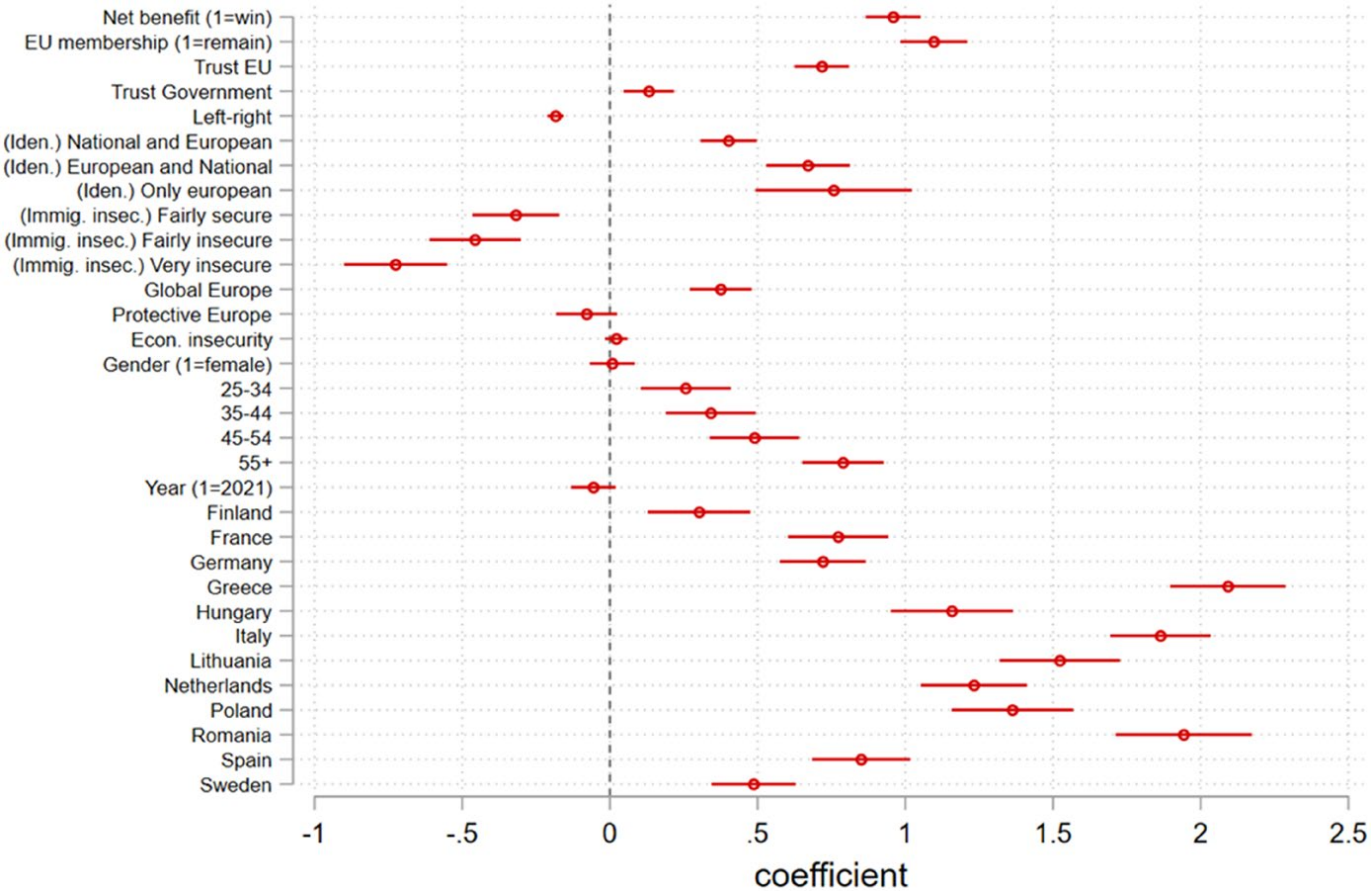
Visualisation of the regression coefficients

Descriptive statistics and distributions of all model variables are displayed in online Appendices II and III. All survey questions and answer items used as indicators in this analysis, as well as response shares, can be inspected in online Appendix IV. It should be noted that the variables were disaggregated into their categories where it is theoretically expected that the relation might not to be linear (*age*), where a variable lacks a central point and has less than 5 categories (*immigration insecurity* and *identity*) or where the variable is purely categorical (*preferred* *model for Europe* and *country*). The plots in the next section omit the following reference categories for each categorical variable: ‘only national’ (*identity*); ‘very secure’ (*immigration insecurity*); ‘Market Europe’ (*preferred model for Europe*); ‘18–24’ (*age*); and ‘Denmark’ (*country*). This model specification means a dummy is calculated for each of the levels of these variables, which is why they are represented as separate correlates in Figs. 2 and 4. Despite this visualisation issue, as we will see, the difference in the magnitude of effect size of net-benefit expectations, welfare protectionism, identity and immigration insecurity is nevertheless clear.

## Results

**Fig. 3:**
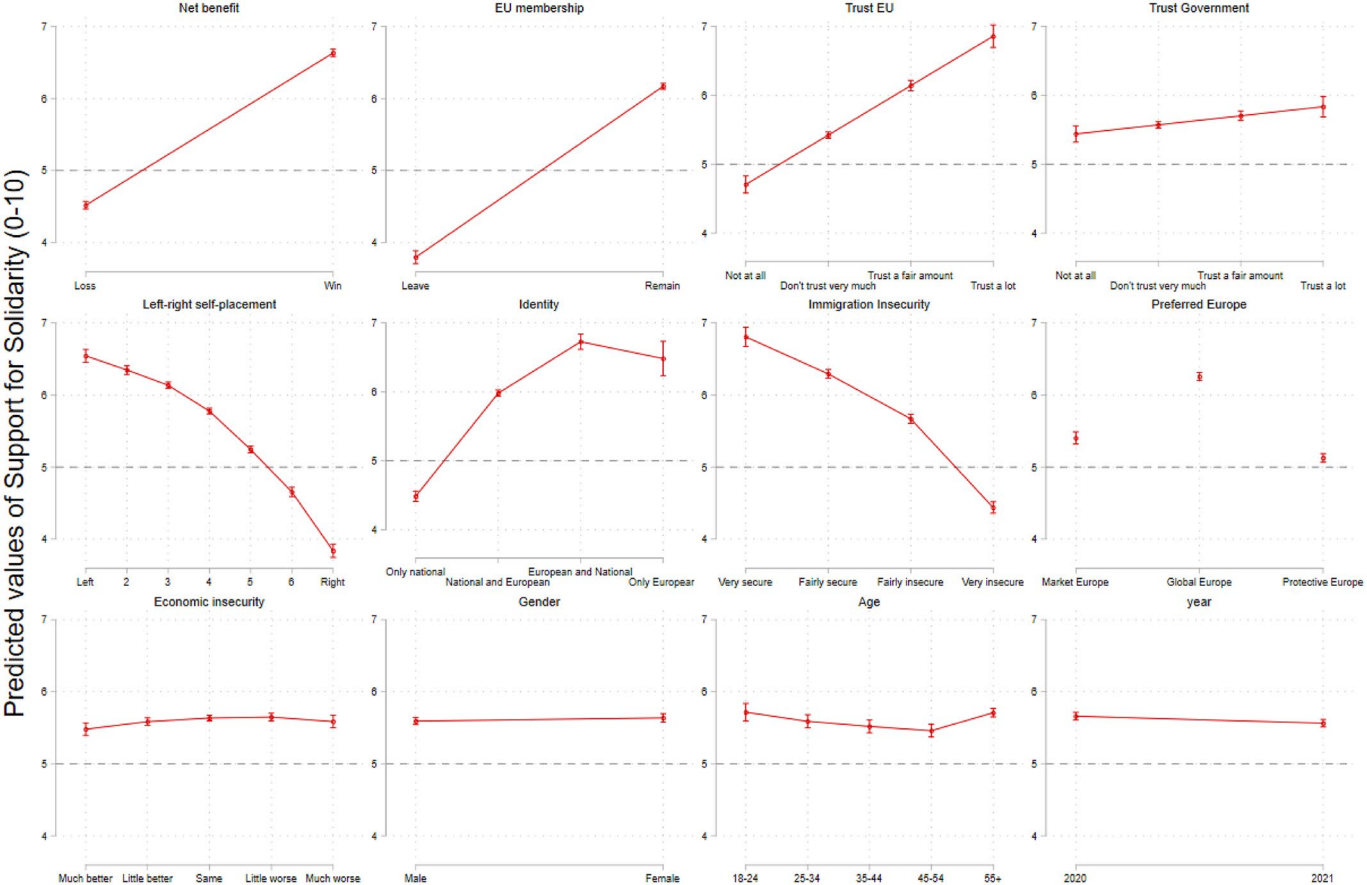
Adjusted linear predictions of support for solidarity

**Fig. 4:**
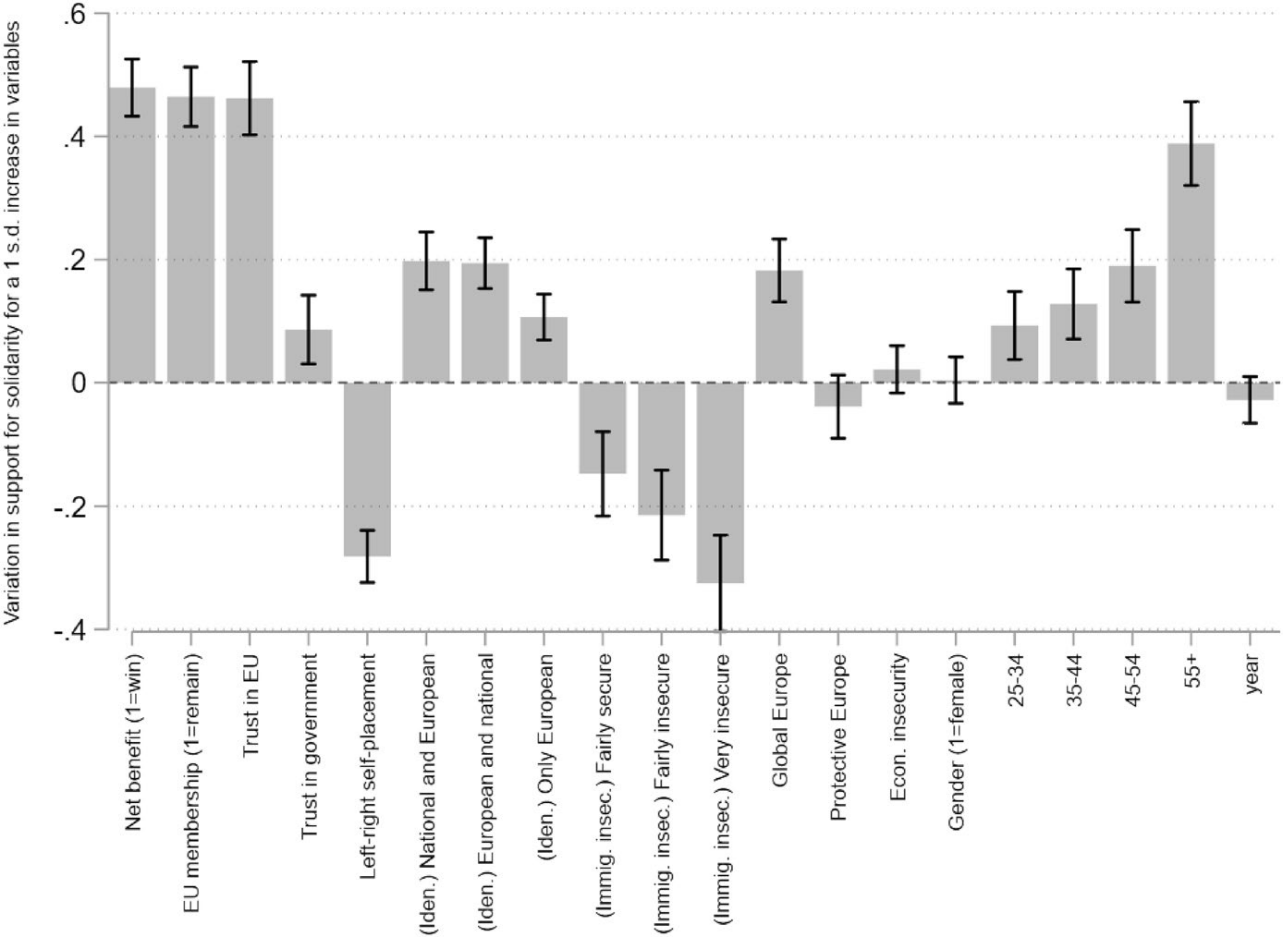
Comparison of standardised regression coefficients in support for solidarity

The analysis results are summarised in three main outputs: Fig. 2 plots regression coefficients; Fig. 3 plots adjusted linear predictions of support for solidarity along the categories of each independent variable (with other variables controlled at mean values); finally, Fig. 4 plots a comparison of the standardised effect size of each correlate in the model, allowing to measure the strength of each set of determinants under analysis (utilitarian, cultural, social). This figure illustrates how much support for solidarity increases or decreases (in its scale, 0–10) for each one additional standard deviation increase in the model variables.

As anticipated, the results fall in line with our theoretical assumption that most determinants identified in the literature correlate with solidarity support to a statistically significant extent, which can be confirmed by a visual inspection of Fig. 2. The regression table can be consulted in online Appendix V. Surprisingly, our data do not support the contention that gender and subjective economic insecurity are correlated with support for European solidarity, unlike suggested by previous studies (e.g. Kleider and Stoeckel 2018 or Baute et al 2019). This suggests that determinants of solidarity are informed primarily by *political* dispositions. Year effects are also non-significant, showing indicator stability between 2020 and 2021.

Concerning H1, the analysis suggests two main conclusions: first, that *trust in EU*, *trust in national governments* and *attitudes towards EU membership* are *positively* correlated with support for EU solidarity. In short, individuals support European solidarity the more they trust their institutions. Second, that the most relevant political attitudes for European solidarity are those directed at the supranational level, even after controlling for trust in domestic governments: the slopes in Fig. 3 for *trust in EU* and *attitudes towards EU membership*, much steeper than that of *trust in national governments*, confirm these variables account for more variation in support for solidarity. The standardised comparison in Fig. 4 further clarifies this contention: *attitudes towards EU membership* and *trust in EU* are two of the three most important correlates of European solidarity, with a noticeable difference to *trust in governments* which, although statistically significant, has a much more limited explanatory potential.

The importance of supranationally informed preferences regarding EU solidarity suggests that citizens evaluate European solidarity as a legitimate solidaric space on its own right. Support for solidarity appears to be an individual expression of political attachment to the supranational community and its institutions, *regardless* of how citizens feel about their national ones. This evidence appears to contradict the so-called *proxy theory* whereby attitudes towards EU affairs would be a projection onto the supranational level of national political predispositions; instead, the strong explanatory role of attitudes concerning the EU polity directly may actually suggest a manifestation of individual preferences to engage in the sharing of resources and risks with the supranational community, as an overlapping but equally legitimate level for solidarity (Baute et al 2019). This appears to confirm H1, i.e. that individual support for EU solidarity is more influenced by an orientation towards the supranational institutional level than the national level.

Concerning H2 on the nature of motivations underlying support for solidarity, we can gather that expectations of *net-benefit*, *national identity* and *immigration insecurity* display a significant (Fig. 2) and important (Fig. 3) correlation with support for EU solidarity. Respondents who expect the country to win from an emergency EU solidarity fund tend to support it more than those who expect the country to lose from said fund. Also, the less one identifies with an exclusively national identity, the more one tends to support solidarity. Concurrently, those who feel more insecure about immigration also tend to oppose solidarity more. These findings, at least at face value, corroborate previous theoretical accounts of self-interest and cosmopolitanism being important predictors for public support for redistribution. Somewhat more surprising is that support for solidarity is not correlated with the preference for a ‘Protective’ Europe, suggesting a social EU agenda upholding European welfare is not a relevant predictor of support for European solidarity.

What is new about this analysis is the comparative assessment of the *strength* of these determinants: the results in Fig. 4 appear to indicate that EU solidarity is more aptly captured by utilitarian expectations from international solidarity than cultural-based explanations emphasising national identity and immigration. While the slopes of these three variables in Fig. 3 suggest all are relevant explanations, in Fig. 4 we ultimately observe that *net-benefit* expectations are, indeed, the most relevant determinant of European solidarity in the dataset. This compares to an evidently lesser effect size of *identity* and *immigration insecurity* coefficients which, although relevant, explain variation in support for European solidarity to a lesser extent.

Circling back to the ideal-type typology, this analysis illustrates the contention that arguments emphasising utilitarian determinants are better able to account for support for EU solidarity than cultural and social arguments, thus confirming H2. It should be noted, however, that this evidence does not back a clear-cut claim against the relevance of cultural explanations, as both material- and cultural-based arguments remain very significantly equipped to empirically and theoretically account for support for solidarity, itself the product of a conjunction of several different factors operating simultaneously. This finding entails important conclusions to European solidarity, particularly at a time when, although nationalism and opposition to immigration were quite present, the salience of EU-led fiscal solidarity and the size of redistributive packages was remarkably high.

**Fig. 5:**
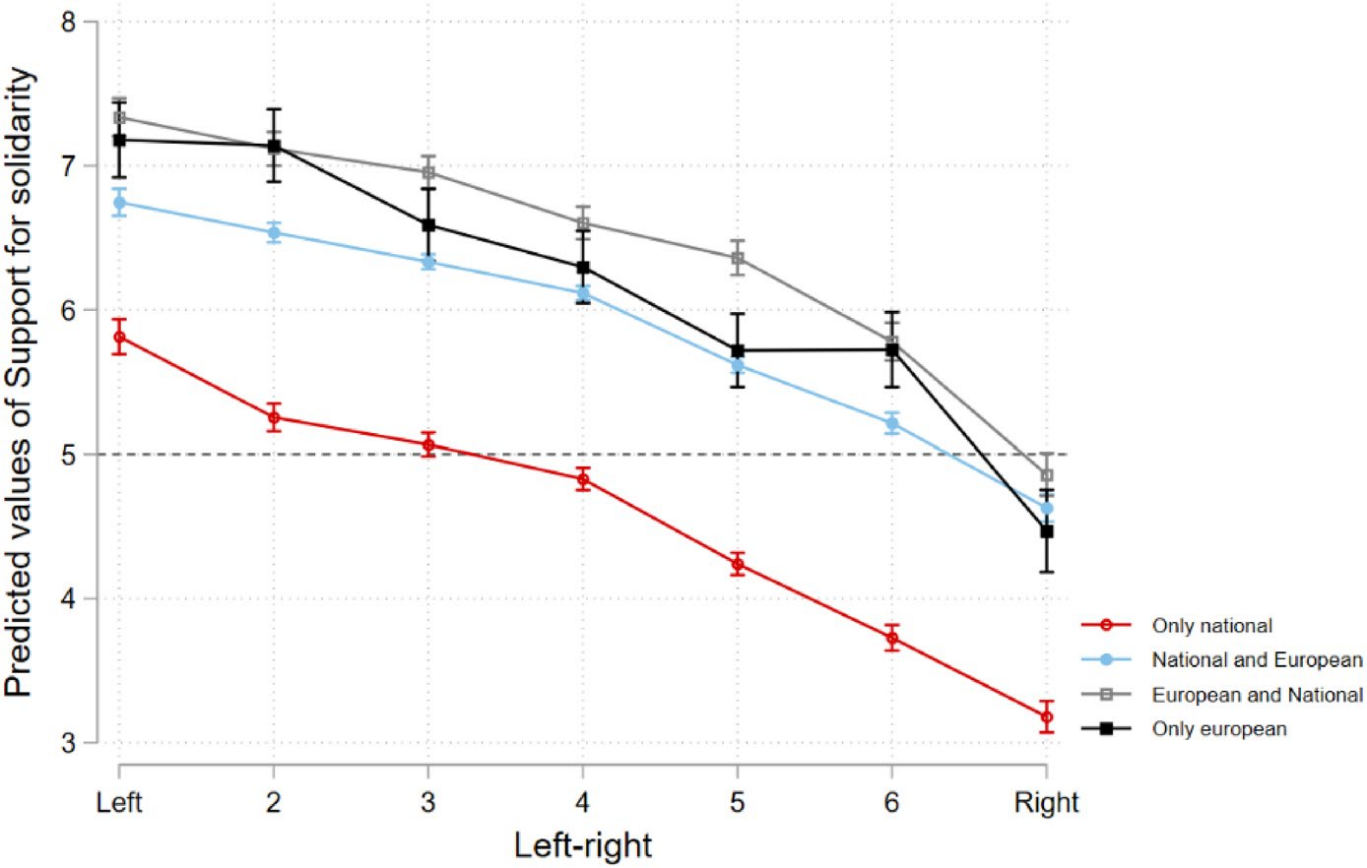
Predicted levels of support for solidarity in different identity subgroups along the left–right scale

**Fig. 6:**
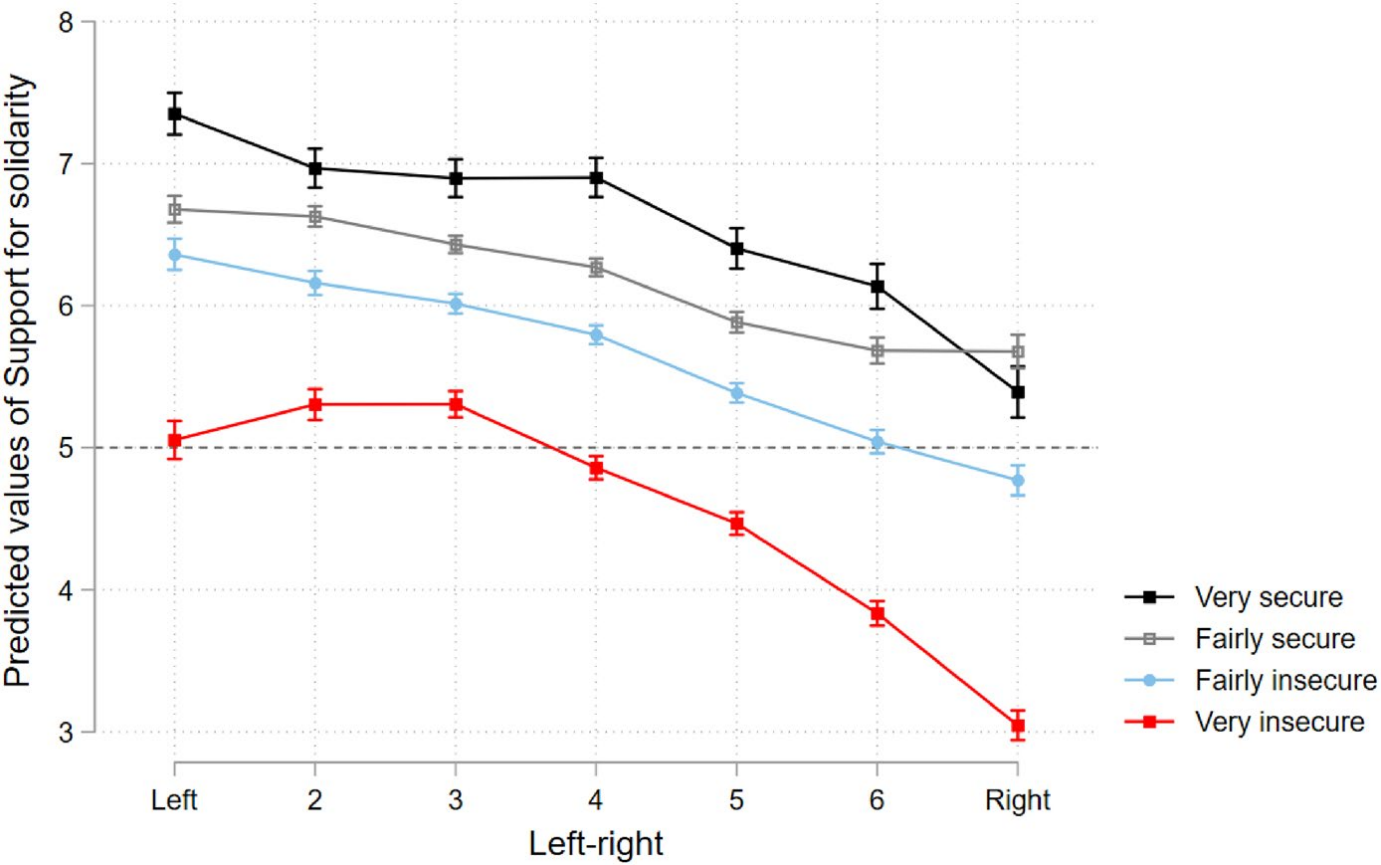
Predicted levels of support for solidarity in different immigration insecurity subgroups along the left–right scale

H3 explored whether solidarity support is better captured by a cultural demarcation/integration divide or a materialist traditional left–right divide. A visual representation of predicted levels of support for solidarity within groups displaying different attitudes towards *identity* and *immigration* are shown in Figs. 5 and 6, respectively. Each of these groups is represented by a slope, and each group’s average support for solidarity is distributed along the levels of the left–right spectrum (as the variable representing the traditional economic divide). If the cultural divide was to be the determinant one, an interpretation would be that the slopes would tend to follow a flat or inverted U shape (*vide* theoretical outline in online Appendix I) and place vertically more distant from each other, meaning variables encapsulating the demarcation/integration divide (spread vertically over the y-axis) would account for more variance than the x-axis (*left–right*). An economic divide would see linear, steeper slopes, with the lines vertically closer together, emphasising that most of the variation is explained by the x-axis (*left–right*) and less by the cultural divide variables spread over the y-axis.

The evidence is mixed: on the one hand, the more one moves to the right, the more opposition is found against EU solidarity. This falls in line with economic divide expectations, at least for those with multiple or only European identities and those who feel less insecure regarding immigration, as the vertical proximity between these groups’ slopes indicates their support for solidarity does not differ much due to their views regarding *identity* and *immigration* but rather on their *left–right* placement. However, two specific groups (those with exclusive national identities and those very insecure about immigration, in red) display much lower levels of support for solidarity than the other groups across the entire left–right spectrum. They stand out due to the vertical distance between their slopes and those of the other groups over the y-axis. This stark divergence between these two groups (more nationalist-oriented) and other groups (more cosmopolitan-oriented) suggests *immigration* and *identity* are important operators concerning support for solidarity, but only among the former.

At the same time, even within nationalist-oriented groups, we find a linear erosion of solidarity support the more an individual identifies with right-wing views, suggesting that left-wing respondents on the national and anti-immigration end are still much more favourable to EU solidarity than those who share the same beliefs on the right. This suggests pro-demarcation far-left individuals might still be more supportive of solidarity than pro-demarcation far-right individuals. Importantly, the correlation strength of *left–right* positions among the less cosmopolitan is much more pronounced regarding identity than immigration.

In short, the evidence appears to be inconclusive regarding the adequacy of one theoretical divide over the other, as the data appear to indicate both are relevant to explain support for solidarity, according to the group in question—an economic left–right divide appears more appropriate over the less nationalistic and more pro-immigration, whereas an economic left–right *and* a cultural demarcation/integration divide appear to operate simultaneously among the more nationalistic and anti-immigration citizens. This appears insufficient to fully confirm H3.

**Fig. 7:**
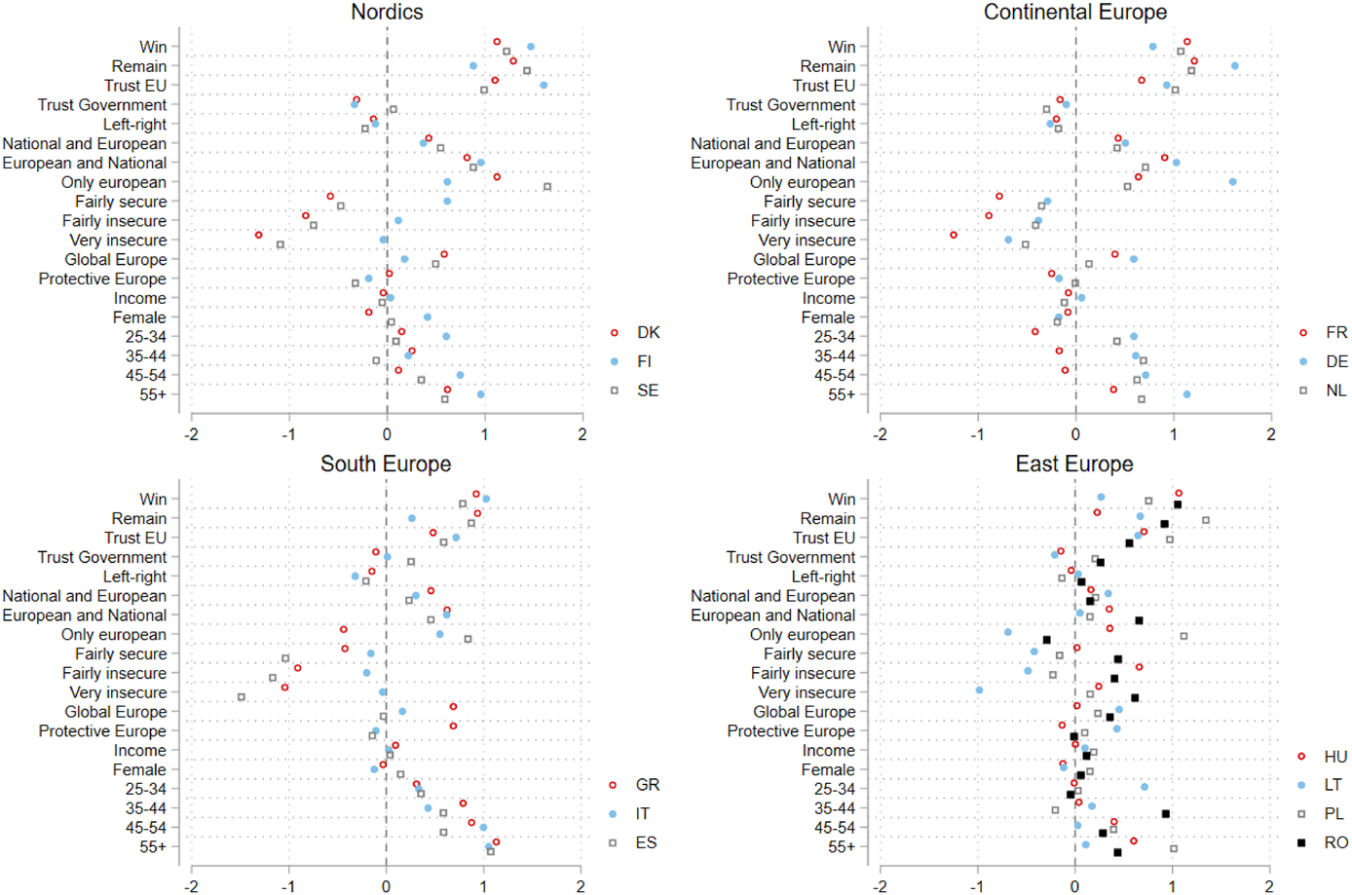
Coefficient estimation by region

Finally, a country-by-country analysis was conducted as a check to the robustness of the data, in Fig. 7. A detailed representation of regression coefficients at the country-level can be found in online Appendix VII. Overall, there are no significant outliers: all countries display a similar trend in coefficient dispersion for every variable, albeit less so for *immigration insecurity* and *identity*, where correlation with support for solidarity differs the most across countries. If we divide the surveyed countries into four regions (Nordics, Continental, South and East Europe), we observe that the four groups display very similar patterns, with the exception of these two variables in Eastern Europe, where they correlate less with support for solidarity than in other regions. However, this is the only major difference across regions in terms of effects of the variables of interest in support for solidarity. In sum, country-level and regional-level results align with the main conclusions of the supranational-level analysis, lending support to the latter.

## Discussion

Variation in support for solidarity is expected to occur primarily across individuals rather than across countries. This implies that preference formation regarding supranational redistribution is informed by subjective, interpersonal dispositions rather than differences in national conditions; under this assumption, even sociotropic views focussing on perceived national economic benefit or loss are only relevant predictors of support for solidarity insofar as they are primarily a reflexion of individual perceptions. This illustrates the propriety of a comparative appraisal of the determinants behind *individual* support for solidarity, particularly at a time of crisis characterised by symmetric pandemic shocks across the Union, the levelling up of solidaristic supranational responses to those shocks, and the instrumental economic nature of redistribution underscoring the post-COVID recovery ahead.

The results in this paper seem to confirm that citizens reason in supranational terms. Attitudes towards EU membership and political trust in EU institutions, understood as a commitment to share risks and resources across a territorially defined political community, are among the stronger determinants of individual solidarity support. This consubstantiates the claim that underlying preferences regarding EU solidarity operate political attitudes directly primarily towards the supranational level. This finding suggests that European solidarity exists as a distinct legitimate space for redistribution in its own right, sustained by a direct polity legitimacy link between individuals and supranational institutions, and not as an extrapolation of domestic political preconditions.

This does not preclude that, despite acknowledging diverse multilevel solidaristic spaces, individuals look for maximisation of self-interested gains. Just like individuals may recognise a national solidaristic community but search for gains towards their own region, it is reasonable to expect this may also happen between national and supranational levels. Our results are consonant with the theoretical claim that the substantive nature of motivations behind European solidarity are better captured by *zweckrational* utilitarian arguments than by cultural or social explanations. The implications of utilitarian motivations behind EU solidarity reflect the fact that EU solidarity, as all modern solidaric spaces encompassing large and heterogenous members, is exposed to fundamental challenges such as fears of lack of reciprocity and moral hazard (Genschel et al 2021).

This finding must be interpreted alongside other intervening factors behind support for solidarity. Indeed, the analysis carried out in this paper was designed to comparatively explore the strength of each correlate in predicting support for solidarity, under the assumption all indicators in the model were relevant predictors to some degree. This stems not only from the well-established theoretical expectation that multiple factors concur in explaining support for solidarity, but also from empirics: there is limited evidence to confirm that the political divides on support for solidarity reflect economic left–right rather than cultural integration/demarcation logics. While the former (typically focussed on redistribution in capitalist societies, with the left-wing supporting it due to an adherence to social fairness and economic equity values, and the right-wing opposing it as an expression of economic interventionism) is useful to determine support for solidarity among the more cosmopolitan, it does not capture views among the more nationalist-inclined citizens who fear immigration—strengthening the case for non-economic reasonings underpinning European solidarity. The caveat of the multivariate cross-sectional design is that it ultimately does not allow to disentangle the complex way in which the intervening variables relate to one another, identifying pathways for further causal identification research on this topic.

In the context of a critical juncture for EU solidarity due the wide-reaching effects of the pandemic and the orientation of citizen preferences towards material advantages from redistribution efforts, the emergence of European instruments for economic redistribution in the post-COVID recovery may prove to be a landmark development in the establishment of a supranational solidaristic space and an important opportunity for EU *bonding* and polity-building. To confirm this, future research would be in order to extricate the evolution of individual preferences towards solidarity and the link between these preferences and success (or failure) of EU financial stimulus packages such as Next Generation EU. New avenues could also be explored concerning the geographical variation of determinants of support for solidarity across member states, as this paper focussed only at the supranational-level; it might also be worthwhile to see how future crises of other types affect salience and motivations underlying EU solidarity, with the current war in Europe providing a novel and probably different scenario underpinning support for EU solidarity. While this contribution cannot realise such a long-term appraisal as it cannot look into the future, it can nonetheless contribute by advancing a typology of theoretical accounts for variation of individual support for European solidarity, which might assist future research on this issue.

## Supplementary Information

Below is the link to the electronic supplementary material.

Supplementary file1 (DOCX 963 kb)
