# Supplementary material for: A supranational solidaristic space? Comparative appraisal of determinants of individual support for European solidarity in the COVID-19 era
Source: Comp Eur Polit. 2023 Apr 7:1–21. Online ahead of print. doi: 10.1057/s41295-023-00345-5 (PMC10081298; doi:10.1057/s41295-023-00345-5)

# Online Appendices

## Online Appendices for “A supranational solidaristic space? Comparative appraisal of determinants of individual support for European solidarity in the COVID-19 era”

## Luís Russo

# Appendix I

Theoretical map

### Utilitarian determinants

The first theoretical group deals with utilitarian motivations for solidarity. This strand of literature emphasizes a rational cost-benefit calculus (Downs, 1957) behind support for EU solidarity, emphasizing *zweckrational* self-interested motives for supporting solidarity. Given that EU solidarity is primarily exercised as transfers of resources between governments, anchoring this calculus is the individual perception on whether one’s own country is a winner or loser of fiscal transfers among member states, i.e., whether one expects their country to receive more resources than it has contributed (net benefit) or, conversely, one expects to their country to contribute with more resources than it has received (net loss). Under this sociotropic assertion, individuals support solidarity to the extent that they consider said solidarity to be aligned with the national interest, subscribing to it conditionally to their expectations on receiving an advantage superior to their contribution (Beaudonnet, 2014, Daniele and Geys, 2015, Kleider and Stoeckel, 2019) - as other authors argue is also the case with other EU redistribution mechanisms such as the European Structural and Investment Funds (Rodríguez-Pose and Dijkstra, 2021).

### Social determinants

A more social-oriented argument states that support for solidarity reflect views on ‘what constitutes a good policy for society as a whole’ instead of maximisation of own economic benefit (Corneo and Grüner, 2002, p. 84). Attitudes would be strongly supported by economic redistribution preferences while also informed by an adherence to the supranational rather than national interest. According to this argument, support for solidarity would be primarily motivated by values about economic fairness and equality to promote a similar level of material welfare across the Union. Generally, acknowledging the extensive interdependence among member states (Lengfeld *et al*, 2015), citizens would support more permanent redistribution mechanisms to mitigate social imbalances within the Union (Fernandes and Rubio, 2012) and more supranational-oriented social policy designs and resource allocation, stressing the need for a more ‘Social Europe’ with which to tackle challenges that face the internal market and the Eurozone as a whole (Vandenbroucke, 2013).

It is posited that citizens who view integration as connected to the enhancement of a European social policy are more inclined to support solidarity transfers among countries as means to implement decent cross-border welfare standards across the Union, while those who prioritise strictly economic policies like strengthening market integration and European competitiveness would be less inclined to do so, ascribing solidarity to realm of Social Europe instead of European economic integration (Baute *et al*, 2019).

### Economic left-right divide

The preference towards a redistributive EU solidarity is also reflexive of traditional economic left-right divides. Left-wing citizens typically support of a more interventionist role of (supranational) government, uphold values of social fairness and are more adept of wealth redistribution to improve welfare standards; hence, they would be generally more willing to support international redistribution and citizens than citizens on the right who oppose supranational economic regulation and intervention (Hooghe and Marks, 1999, Bechtel *et al*, 2012, Beaudonnet, 2014; Daniele and Geys, 2015; Kuhn et al, 2018).

### Protective determinants

Some other literature emphasizes cultural motivations as key preconditions for support towards EU solidarity. The main motivations presiding support for solidarity would not be chiefly linked to the amelioration of material well-being but rather attached to the individual’s national identification. In other words, allocation lines would reflect (the existence or absence of) cultural integration-demarcation divides, and not so much convergence of diverging socio-economic realities (Kuhn *et al*, 2018, Kuhn and Stoeckel, 2017, Bauhr and Charron 2021, Kleider and Stoeckel, 2018, Baute *et al*, 2019; see also Kriesi *et al*, 2012).

This argument reflects on the absence of a European identity (De Wilde and Trenz, 2012) and resonates with postfunctionalist literature on the politicisation of EU issues domestically and its enabling of an increasing contestation of EU integration as a result of the mismatch between intromission of EU policies in domestic affairs and a perceived threat to individual national identities (e.g., Carey, 2002, McLaren, 2004, Hooghe and Marks, 2009). Using the EU sovereign debt crisis as an illustration, some authors point that bailout transfer were unpopular in receiving countries, where publics viewed conditionality attached to international transfers as a relinquishment of national sovereignty (Verney, 2017), and in contributing countries, where publics viewed support as an imposition to help profligate *others* rather than a contribution to mitigate adversity in afflicted community peers (Kuhn and Stoeckel, 2014). This escapes a logic of domestic net benefit/loss or supranational welfare improvement, patent in material explanations, and emphasizes the importance of territorial identity as the main driver behind support for solidarity, pitting ‘nationalist sentiments versus greater cosmopolitan affinity’ (Bechtel *et al*, 2014, p. 835).

As a result, an exclusive identification with national identity and scepticism of other EU nationals (Hooghe and Marks, 2009) would be linked to lower support for EU solidarity (Bechtel *et al*, 2014, [Daniele and Geys, 2015](https://www.tandfonline.com/doi/pdf/10.1080/13501763.2014.988639?casa_token=KpiqElsZCI8AAAAA:hveYHdoUSlfVbcekZDmguJwrFX4RByNfkkhOOWdtRMPXzRb-xwbdQCzVgqcojo1dqavsBUaqJUba), Kleider and Stoeckel, 2018). There is some evidence that this demarcation is reflected in a cultural political divide pitting higher-earning, culturally open mainstream party voters against those lower-income voters who, feeling that an expansion of European integration leaves them more exposed to international economic competition and encapsulates a threat to their social entitlements, would mobilise their political support towards challenger parties on the radical left- and right-wing championing a restoration of domestic sovereignty, immigration controls and domestic welfare protection (Kriesi *et al*, 2012). Taking this theoretical framework into support for European redistribution, while extremist pro-demarcation parties at both ends of the left-right spectrum would view their legitimate solidaric community at the nation-state level, support for supranational solidarity would then fall to more cosmopolitan centrist pro-integration parties (Kriesi *et al*, 2012, Bechtel *et al*, 2014, Hooghe and Marks, 2017).

### Cosmopolitan determinants

In an inverse fashion from those who oppose solidarity as they wish to protect their national ingroup, the literature on cultural determinants behind solidarity suggests that those less identifying exclusively with their national identities are more likely to support solidarity, as their position is informed by a cultural attachment to different, overlapping territorial identities; this enables them to perceive solidarity at the supranational level as a legitimate endeavour (Sniderman *et al*, 2004, Kuhn et al, 2018, Verhaegen, 2017). The literature refers to these multiple attachments as *cosmopolitanism*, suggesting it ‘increases generosity towards other Europeans and support for international redistribution even when controlling for self-interest, support for national redistribution, concern for others and political ideology’ (Kuhn *et al*, 2018, p. 1759). This attachment to multiple identities differentiates it from protective determinants to the extent that they subscribe to one or more political communities, but shares with it the theoretical expectation that it is noneconomic cultural dispositions such as altruism and cosmopolitanism that correlate with solidarity (e.g. [Bechtel *et al*, 2014](https://onlinelibrary.wiley.com/doi/full/10.1111/ajps.12079), Kuhn *et al.*, 2018).

**References**

*Bauhr, M. and Charron, N. (2021) ‘Stand together or alone? Public support for European economic solidarity during the Covid-19 pandemic’, European Societies, 23(4), pp. 533–561.*

*Baute, S., Abts, K. and Meuleman, B. (2019) ‘Public Support for European Solidarity: Between Euroscepticism and EU Agenda Preferences?’, Journal of Common Market Studies, 57(3), pp. 533–550.*

*Beaudonnet, L. (2014) ‘Take One for the Team? A Study of the Individual Bases for European Solidarity in Times of Crisis’. Paper presented at the 10th Biennial Conference of the European Community Studies Association - Canada, Montreal, 8–10 May.*

*Bechtel, M.M., Hainmueller, J. and Margalit, Y. (2014) ‘Preferences for International Redistribution: The Divide over the Eurozone Bailouts’, American Journal of Political Science, 58(4), pp. 835–856.*

*Carey, S. (2002) ‘Undivided Loyalties: Is National Identity an Obstacle to European Integration?’, European Union Politics, 3(4), pp. 387–413.*

*Corneo, G. and Grüner, H.P. (2002) ‘Individual preferences for political redistribution’, Journal of Public Economics, 83(1), pp. 83–107.*

*Daniele, G. and Geys, B. (2015) ‘Public support for European fiscal integration in times of crisis’, Journal of European Public Policy, 22(5), pp. 650–670.*

*Fernandes, S. and Rubio, E. (2012) ‘Solidarity Within the Eurozone: How Much, What for, for How Long?’*

*Hooghe, L. and Marks, G. (1999) ‘The Making of a Polity: The Struggle over European integration’. In: Kitschelt H., Lange P., Marks G., et al. (eds) Continuity and Change in Contemporary Capitalism. Cambridge: Cambridge University Press, pp. 70–79.*

*Hooghe, L. and Marks, G. (2009) ‘A Postfunctionalist Theory of European Integration: From Permissive Consensus to Constraining Dissensus’, British Journal of Political Science, 39(1), pp. 1–23.*

*Hooghe, L. and Marks, G. (2017): ‘Cleavage theory meets Europe’s crises: Lipset, Rokkan, and the transnational cleavage’, Journal of European Public Policy*

*Kleider, H. and Stoeckel, F. (2018) ‘The politics of international redistribution: Explaining public support for fiscal transfers in the EU’, European Journal of Political Research, 58(1), pp. 4–29.*

*Kuhn, T., Solaz, H. and van Elsas, E.J. (2018) ‘Practising what you preach: how cosmopolitanism promotes willingness to redistribute across the European Union’, Journal of European Public Policy, 25(12), pp. 1759–1778.*

*Kuhn, T. and Stoeckel, F. (2014) ‘When European integration becomes costly: the euro crisis and public support for European economic governance’, Journal of European Public Policy, 21(4), pp. 624–641.*

*Kriesi, H., Grande, E., Dolezal, M., Helbling, M., Höglinger, D., Hutter, S., Wüest, B. (2012) Political Conflict in Western Europe. Cambridge University Press.*

*Lengfeld, H., Schmidt, S. and Häuberer, J. (2015) Is There a European Solidarity? Attitudes Towards Fiscal Assistance for Debt-Ridden European Union Member States. Rochester, NY: Social Science Research Network.*

*McLaren, L.M. (2002) ‘Public Support for the European Union: Cost/Benefit Analysis or Perceived Cultural Threat?’, The Journal of Politics, 64(2), pp. 551–566.*

*Rodríguez-Pose, A. and Dijkstra, L. (2021) ‘Does cohesion policy reduce EU discontent and Euroscepticism?’, Regional Studies, 55(2), pp. 354–369.*

*Sniderman, P.M., Hagendoorn, L. and Prior, M. (2004) ‘Predisposing Factors and Situational Triggers: Exclusionary Reactions to Immigrant Minorities’, The American Political Science Review, 98(1), pp. 35–49.*

*Vandenbroucke, F. (2013) ‘Why we need a European Social Union’, Reflets et perspectives de la vie economique, (2), pp. 97–112.*

*Verhaegen, S. (2018) ‘What to expect from European identity? Explaining support for solidarity in times of crisis’, Comparative European Politics, 16(5), pp. 871–904.*

*Verney, S. (2017). Losing Loyalty: The Rise of Polity Euroscepticism in Southern Europe. In Leruth B., Startin N., Usherwood S. (Eds.), The Routledge Handbook of Euroscepticism. London: Routledge*

*de Wilde, P. and Trenz, H.-J. (2012) ‘Denouncing European integration: Euroscepticism as polity contestation’, European Journal of Social Theory, 15(4), pp. 537–554.*

# Appendix II

Table 1: Descriptive statistics of model variables

**Descriptive Statistics**

| Variable | Obs | Mean | Std. Dev. | Scale Min | Scale Max |
| --- | --- | --- | --- | --- | --- |
| Support for solidarity | 36 584 | 5.427 | 3.043 | 0 | 10 |
| Net benefit | 28 544 | .49 | .5 | 0 | 1 |
| EU membership | 32 516 | .726 | .446 | 0 | 1 |
| Trust in EU | 40 905 | 2.163 | .664 | 1 | 4 |
| Left-right placement | 33 521 | 3.95 | 1.542 | 1 | 7 |
| National identity | 40 604 | 1.72 | .688 | 1 | 4 |
| Economic position | 40 570 | 3.008 | .993 | 1 | 5 |
| Gender | 43 372 | .529 | .499 | 0 | 1 |
| Age group | 43 372 | 3.674 | 1.357 | 1 | 5 |
| Preferred Europe | 37 010 | 2.24 | .736 | 1 | 3 |
| Trust in national government | 42 315 | 2.23 | .657 | 1 | 4 |
|  | | | | | |

Appendix III


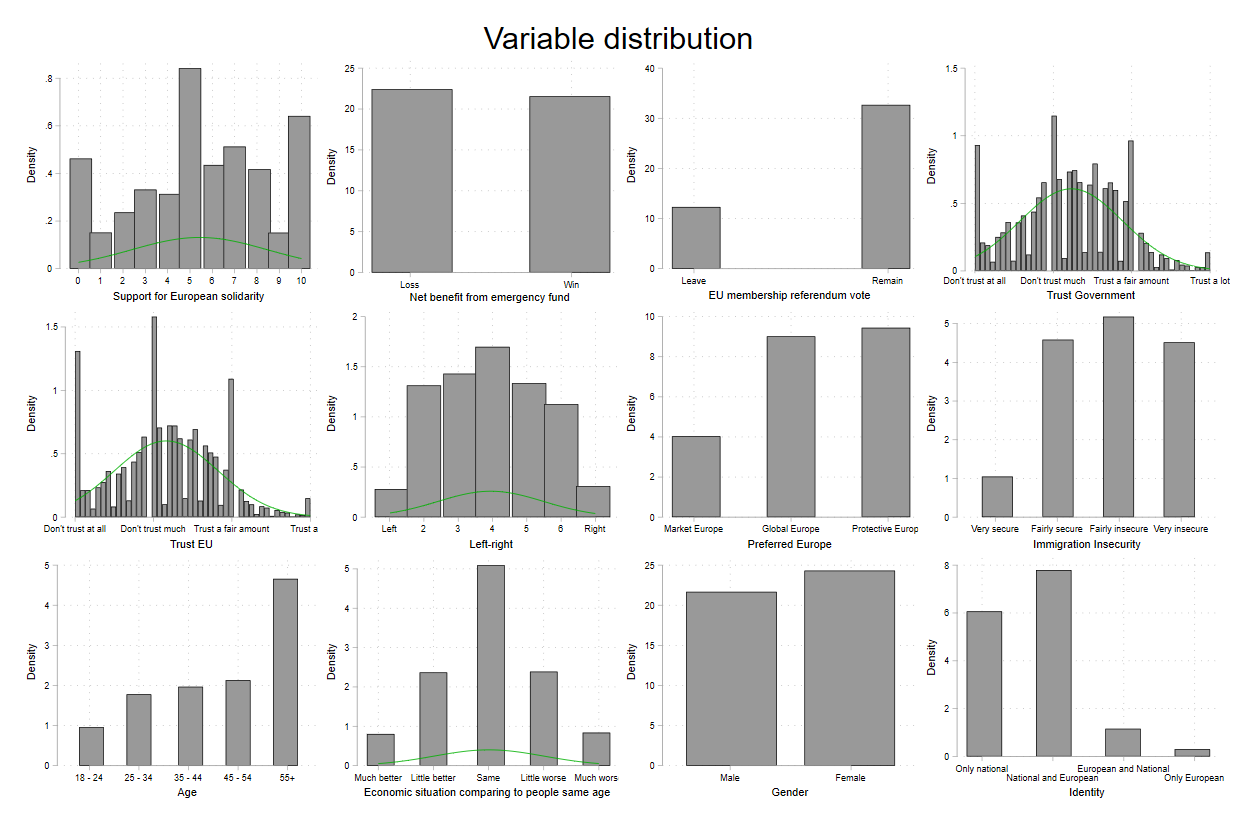
*Figure 8: Distribution of dependent and independent variables*

Appendix IV

Table 2: Survey questions and answers (by order of appearance in the survey)

| **Variable in the model** | **Questions and possible answer items** | **Year** | |
| --- | --- | --- | --- |
|  |  | **2020** | **2021** |
| **Total respondents** | | **19628** | **19575** |
| **Identity** | **Q4. Do you see yourself as...?** | | |
|  | [nationality] only | 35% | 34% |
|  | [nationality] and European | 51% | 50% |
|  | European and [nationality] | 7% | 8% |
|  | European only | 2% | 2% |
|  | None of these | 4% | 4% |
|  | Don't know | 2% | 2% |
| **EU membership** | **Q9. If there was a referendum on membership of the European Union, how would you vote?** | | |
|  | I would vote to remain a member of the European Union | 61% | 63% |
|  | I would vote to leave the European Union | 22% | 20% |
|  | I would not vote | 5% | 6% |
|  | Don't know | 12% | 11% |
| **Preferred Europe** | **Q13. Please tell us in which Europe you would prefer to live** | | |
|  | A market Europe that stresses economic integration, market competition and fiscal discipline | 16% | 16% |
|  | A global Europe that acts as a leader on climate, human rights and global peace | 31% | 33% |
|  | A protective Europe that defends the European way of life and welfare against internal and external threats | 40% | 39% |
|  | None of the above | 6% | 5% |
|  | Don't know | 7% | 7% |
| **Immigration insecurity** | **Q14b_10. Immigration** | | |
|  | Very secure | 6% | 6% |
|  | Fairly secure | 27% | 27% |
|  | Fairly insecure | 33% | 32% |
|  | Very insecure | 26% | 27% |
|  | Don't know enough to say | 7% | 8% |
|  | Net: secure | 34% | 33% |
|  | Net: insecure | 59% | 59% |
| **Trust in government** | **How much do you trust your national government to make things better in the following areas? (Please select one option on each row)** | | |
|  | *(For each of the questions below, respondents could choose one of the following answer items. The percentages are the averages for each answer item across the 11 policy areas)* | | |
|  | Trust a lot | 6% | 6% |
|  | Trust a fair amount | 33% | 31% |
|  | Do not trust very much | 34% | 34% |
|  | Do not trust at all | 21% | 24% |
|  | Don't know enough to say | 6% | 7% |
|  | Net: trust | 39% | 36% |
|  | Net: don’t trust | 55% | 57% |
|  | **Q15_1. The economic situation** | | |
|  | **Q15_2. Climate change** | | |
|  | **Q15_3. Military defence** | | |
|  | **Q15_4. Protection against terrorism** | | |
|  | **Q15_5. Protection against crime** | | |
|  | **Q15_6. Food standards** | | |
|  | **Q15_7. Employment opportunities in your area** | | |
|  | **Q15_8. Your own financial situation** | | |
|  | **Q15_9. Healthcare** | | |
|  | **Q15_10. Immigration** | | |
|  | **Q15_11. Pensions** | | |
| **Trust in EU** | **And how much do you trust the European Union to make things better in the following areas? (Please select one option on each row)** | | |
|  | *(For each of the same questions above, respondents could choose one of the following answer items. The percentages are the averages for each answer item across the 11 policy areas)* | | |
|  | Trust a lot | 5% | 5% |
|  | Trust a fair amount | 29% | 29% |
|  | Do not trust very much | 35% | 35% |
|  | Do not trust at all | 22% | 21% |
|  | Don't know enough to say | 9% | 10% |
|  | Net: trust | 34% | 34% |
|  | Net: don’t trust | 57% | 56% |
| **Support for European solidarity** | **Q18. Some people think that the member states of the European Union should mostly spend their resources on their own countries and the welfare of their own people. Other people think that the member states of the European Union should pool their resources and spend them on all countries and all people across the whole of the European Union. What about you – on a scale of 0 to 10, where 0 means 'Spending resources only on your own country and your own people', and 10 means 'Spending resources equally on all countries and all people in the European Union', where would you put your opinion?** | | |
|  | 0 - Spend resources only on own country and own people [0] | 8% | 9% |
|  | 1 [1] | 3% | 3% |
|  | 2 [2] | 5% | 5% |
|  | 3 [3] | 6% | 7% |
|  | 4 [4] | 6% | 7% |
|  | 5 [5] | 17% | 18% |
|  | 6 [6] | 9% | 9% |
|  | 7 [7] | 11% | 11% |
|  | 8 [8] | 9% | 9% |
|  | 9 [9] | 4% | 3% |
|  | 10 - Spend resources equally on all countries and all people in the European Union [10] | 15% | 15% |
|  | Don't know | 7% | 6% |
|  | Mean excluding don’t know | 5,61 | 5,58 |
| **Net benefit** | **Q43. If there was a large EU-wide emergency fund for EU countries to draw on when they faced a variety of different types of crisis. Do you think that, over a long period of time, [country] would ultimately be an overall…** | | |
|  | Winner: the help received from this fund by [country] would be higher than the resources put in by it (the national net-balance is positive) | 38% | 37% |
|  | Loser: the resources put into this fund by [country] would be higher than the help received by it (the national net-balance is negative) | 35% | 36% |
|  | Don't know | 27% | 27% |
| **Economic insecurity** | **Q61. Thinking about whether you are better or worse off economically or about the same as other people your age in [country], where would you place yourself?** | | |
|  | I am much better off than most other people my age | 5% | 6% |
|  | I am a little better off than most other people my age | 22% | 22% |
|  | I am no better or worse off than most other people my age | 43% | 42% |
|  | I am a little worse off than most other people my age | 16% | 16% |
|  | I am much worse off than most other people my age | 7% | 8% |
|  | Don't know | 7% | 6% |
|  | Net: better | 27% | 28% |
|  | Net: worse | 23% | 23% |
| **Left-right self-placement** | **Q62. Some people talk about 'left', 'right' and 'centre' to describe parties and politicians. With this in mind, where would you place yourself on this scale?** | | |
|  | Very left-wing | 3% | 3% |
|  | Fairly left-wing | 12% | 12% |
|  | Slightly left-of-centre | 14% | 14% |
|  | Centre | 18% | 18% |
|  | Slightly right-of-centre | 13% | 14% |
|  | Fairly right-wing | 12% | 12% |
|  | Very right-wing | 3% | 3% |
|  | Don't know | 20% | 18% |
|  | Prefer not to say | 5% | 7% |
|  | Net: Left | 15% | 15% |
|  | Net: Centre | 46% | 45% |
|  | Net: Right | 14% | 16% |
| **Age** | 18 - 24 | 9% | 10% |
|  | 25 - 34 | 16% | 16% |
|  | 35 - 44 | 16% | 16% |
|  | 45 - 54 | 17% | 17% |
|  | 55+ | 41% | 41% |
| **Gender** | Male | 48% | 48% |
|  | Female | 52% | 52% |

Appendix V

*Table 4: Fixed effects model on correlates of support for EU solidarity*

| Support for solidarity | Coef. | | St.Err. | t-value | | p-value | [95% Conf | | Interval] | | Sig |
| --- | --- | --- | --- | --- | --- | --- | --- | --- | --- | --- | --- |
| Net-benefit (1=win) | .959 | | .047 | 20.19 | | 0 | .866 | | 1.052 | | *** |
| EU membership (1=remain) | 1.097 | | .058 | 18.86 | | 0 | .983 | | 1.211 | | *** |
| Trust in EU | .717 | | .047 | 15.23 | | 0 | .625 | | .81 | | *** |
| Trust in government | .132 | | .044 | 3.03 | | .002 | .046 | | .217 | | *** |
| Left-right self-placement  **Identity** | -.184 | | .014 | -13.06 | | 0 | -.212 | | -.156 | | *** |
| National only | 0 | | . | . | | . | . | | . | |  |
| National and European | .402 | | .049 | 8.25 | | 0 | .306 | | .497 | | *** |
| European and national | .67 | | .072 | 9.26 | | 0 | .528 | | .812 | | *** |
| European only  **Immigration insecurity** | .758 | | .135 | 5.61 | | 0 | .493 | | 1.022 | | *** |
| Very secure | 0 | | . | . | | . | . | | . | |  |
| Fairly secure | -.319 | | .075 | -4.25 | | 0 | -.466 | | -.172 | | *** |
| Fairly insecure | -.457 | | .079 | -5.79 | | 0 | -.612 | | -.302 | | *** |
| Very insecure  **Preferred Europe** | -.726 | | .089 | -8.15 | | 0 | -.9 | | -.551 | | *** |
| Market Europe | 0 | | . | . | | . | . | | . | |  |
| Global Europe | .375 | | .054 | 6.99 | | 0 | .27 | | .48 | | *** |
| Protective Europe | -.079 | | .053 | -1.49 | | .136 | -.183 | | .025 | |  |
| Subjective economic insecurity | .021 | | .02 | 1.10 | | .271 | -.017 | | .06 | |  |
| Gender (1=female)  **Age** | .008 | | .039 | 0.21 | | .837 | -.068 | | .084 | |  |
| 18 – 24 years old | 0 | | . | . | | . | . | | . | |  |
| 25 – 34 years old | .256 | | .078 | 3.30 | | .001 | .104 | | .408 | | *** |
| 35 – 44 years old | .341 | | .078 | 4.40 | | 0 | .189 | | .494 | | *** |
| 45 – 54 years old | .49 | | .078 | 6.31 | | 0 | .338 | | .641 | | *** |
| 55+ years old | .789 | | .07 | 11.20 | | 0 | .651 | | .927 | | *** |
| Year | -.056 | | .039 | -1.45 | | .146 | -.132 | | .019 | |  |
| Denmark | 0 | | . | . | | . | . | | . | |  |
| Finland | .302 | | .089 | 3.40 | | .001 | .128 | | .476 | | *** |
| France | .773 | | .087 | 8.92 | | 0 | .603 | | .942 | | *** |
| Germany | .721 | | .074 | 9.71 | | 0 | .575 | | .866 | | *** |
| Greece | 2.092 | | .1 | 20.95 | | 0 | 1.896 | | 2.288 | | *** |
| Hungary | 1.158 | | .106 | 10.97 | | 0 | .951 | | 1.364 | | *** |
| Italy | 1.864 | | .087 | 21.53 | | 0 | 1.694 | | 2.033 | | *** |
| Lithuania | 1.523 | | .104 | 14.62 | | 0 | 1.319 | | 1.727 | | *** |
| Netherlands | 1.232 | | .092 | 13.42 | | 0 | 1.052 | | 1.412 | | *** |
| Poland | 1.362 | | .105 | 13.00 | | 0 | 1.157 | | 1.568 | | *** |
| Romania | 1.942 | | .118 | 16.49 | | 0 | 1.711 | | 2.173 | | *** |
| Spain | .85 | | .085 | 10.05 | | 0 | .685 | | 1.016 | | *** |
| Sweden | .486 | | .073 | 6.68 | | 0 | .344 | | .629 | | *** |
| Constant | 114.946 | | 77.935 | 1.47 | | .14 | -37.814 | | 267.706 | |  |
|  | | | | | | | | | | | |
| Mean dependent var | | 5.612 | | | SD dependent var | | | 2.992 | |  |  |
| R-squared | | 0.295 | | | Number of obs | | | 18062.000 | |  |  |
| F-test | | 253.277 | | | Prob > F | | | 0.000 | |  |  |
| Akaike crit. (AIC) | | 84590.566 | | | Bayesian crit. (BIC) | | | 84848.017 | |  |  |
| **** p<.01, ** p<.05, * p<.1* | | | | | | | | | | | |
|  | | | | | | | | | | | |
|  | | | | | | | | | | | |

Appendix VI

*Table 5: Left-right self-placement country means and standard deviation*

Appendix VII

*Figures 9 to 18: regression coefficients of model variables for each country*


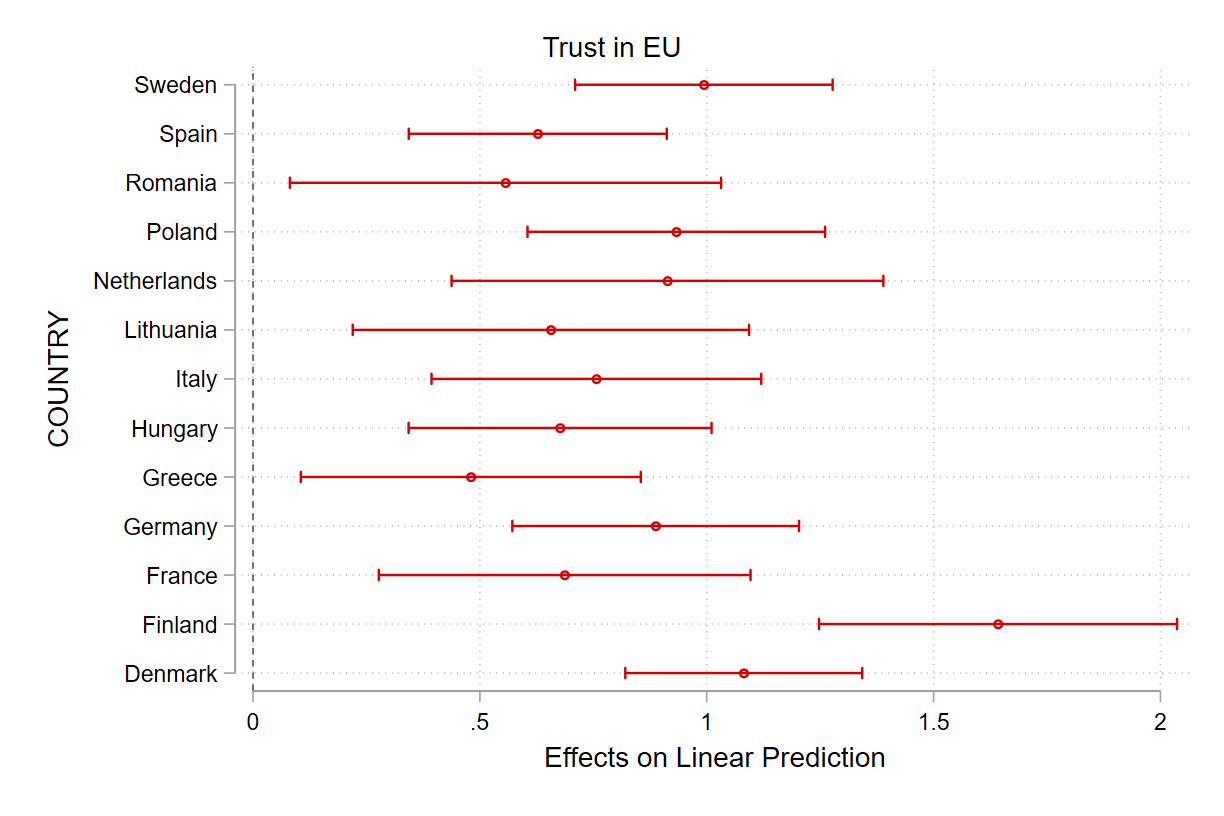

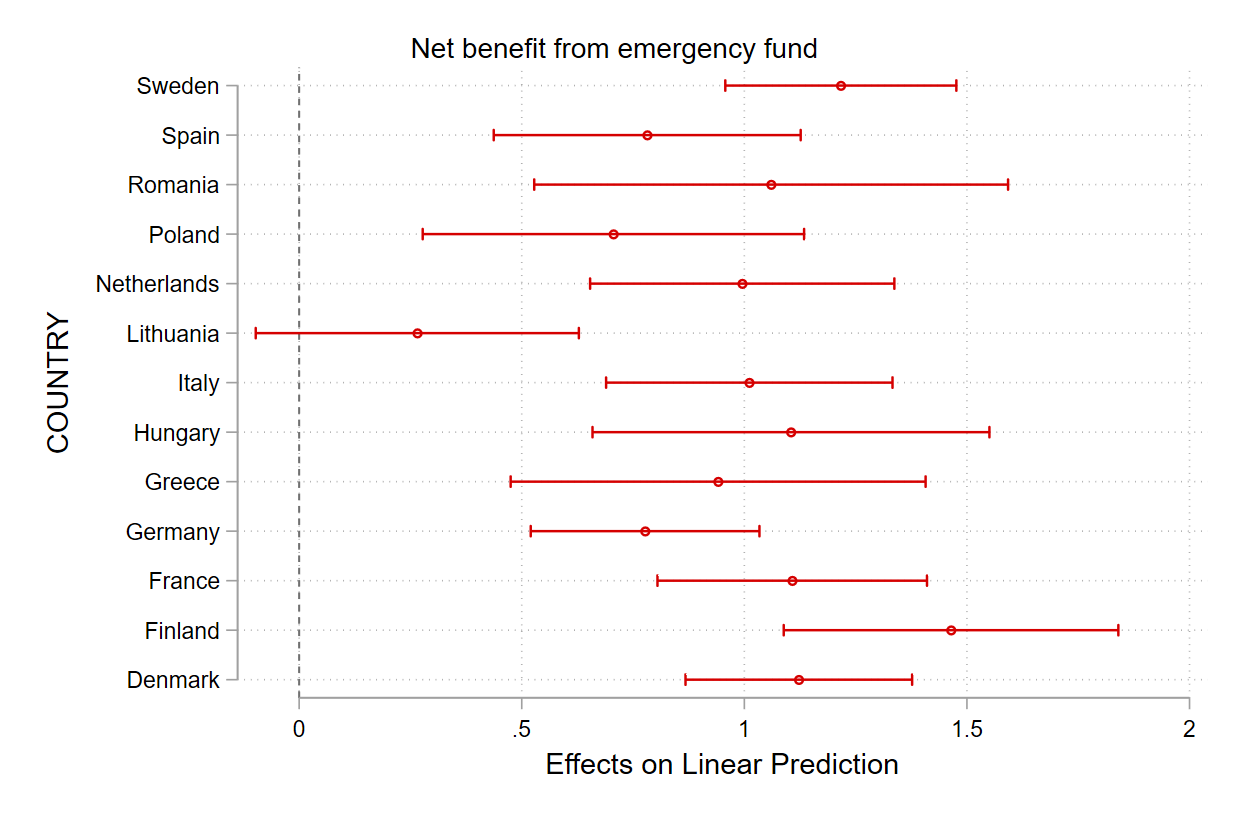

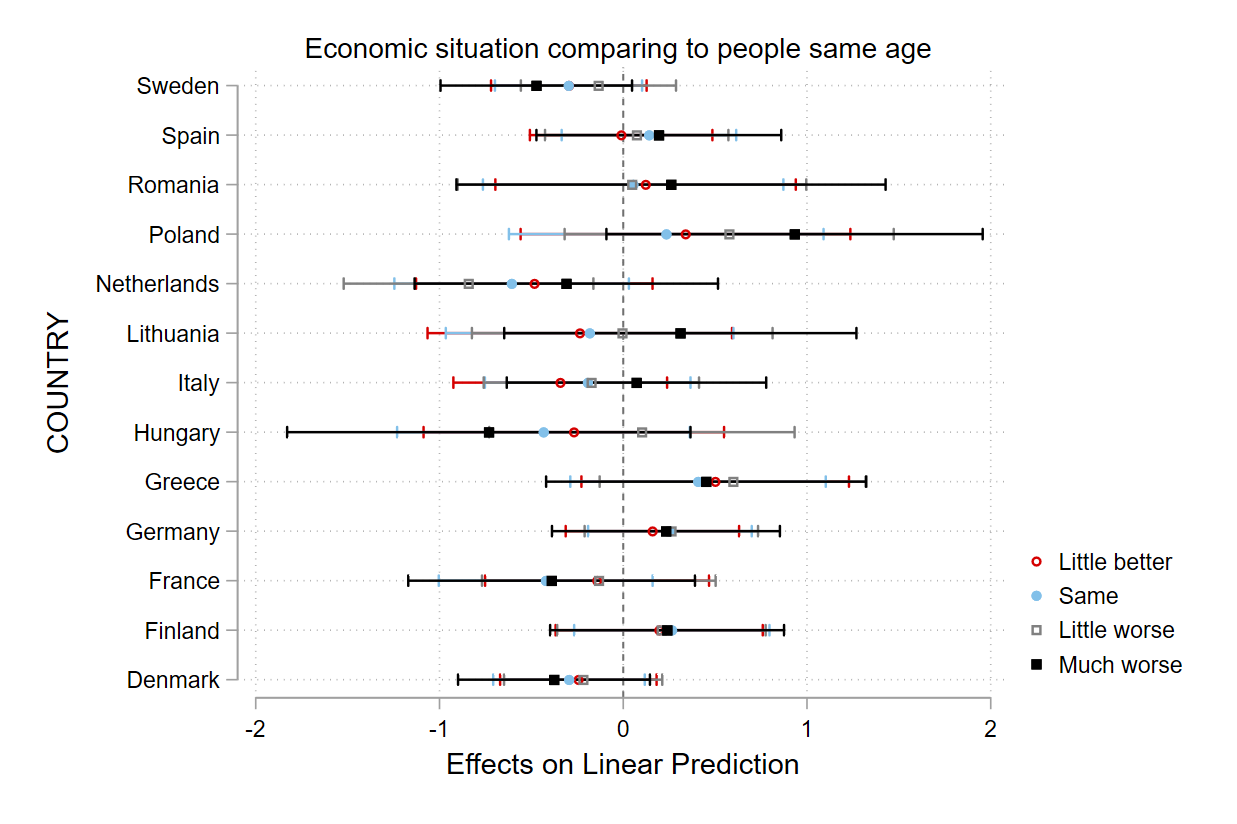

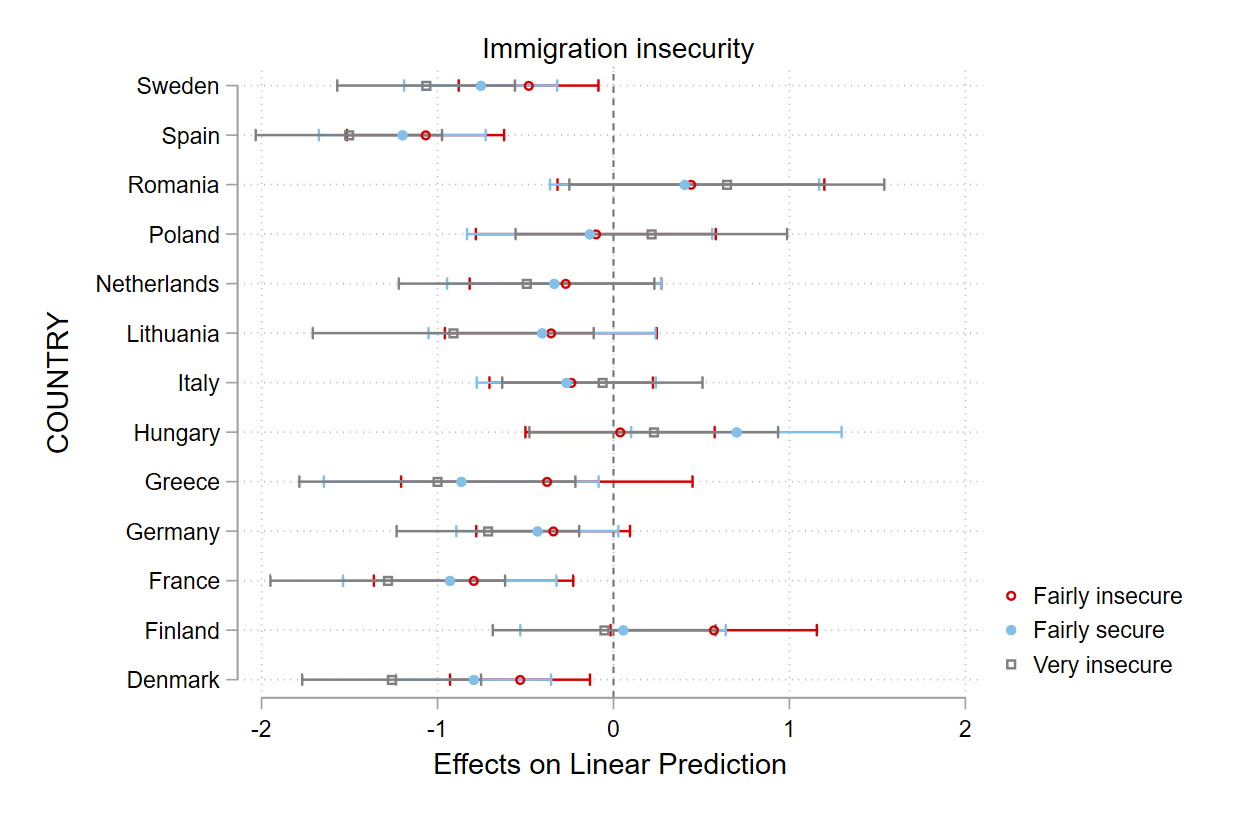

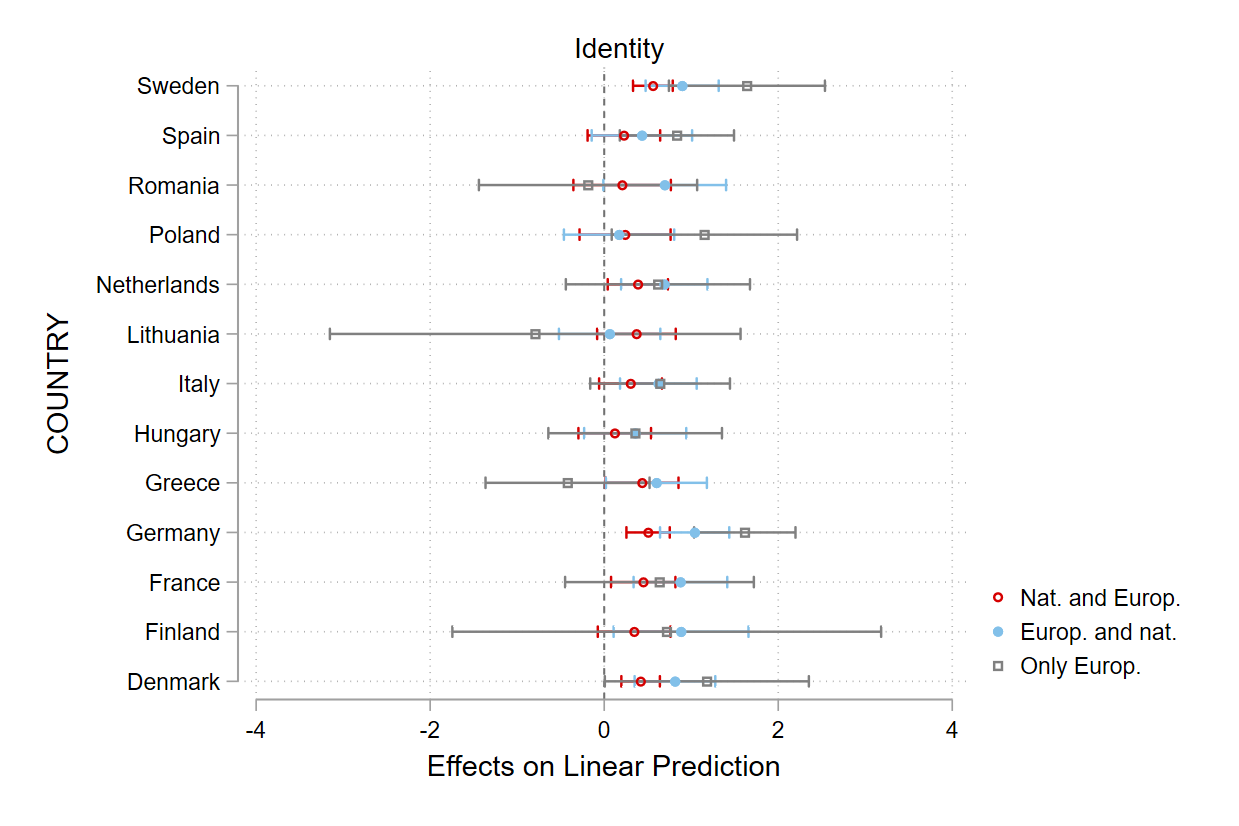


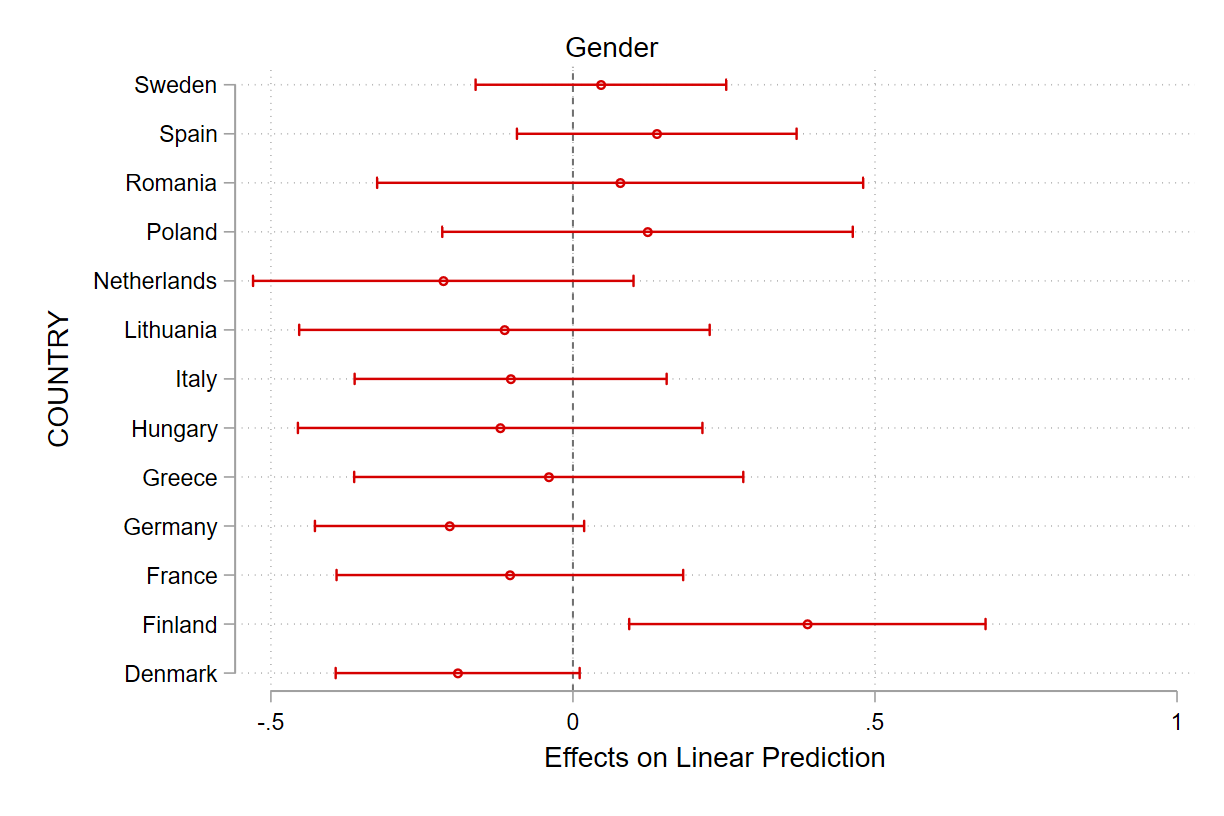

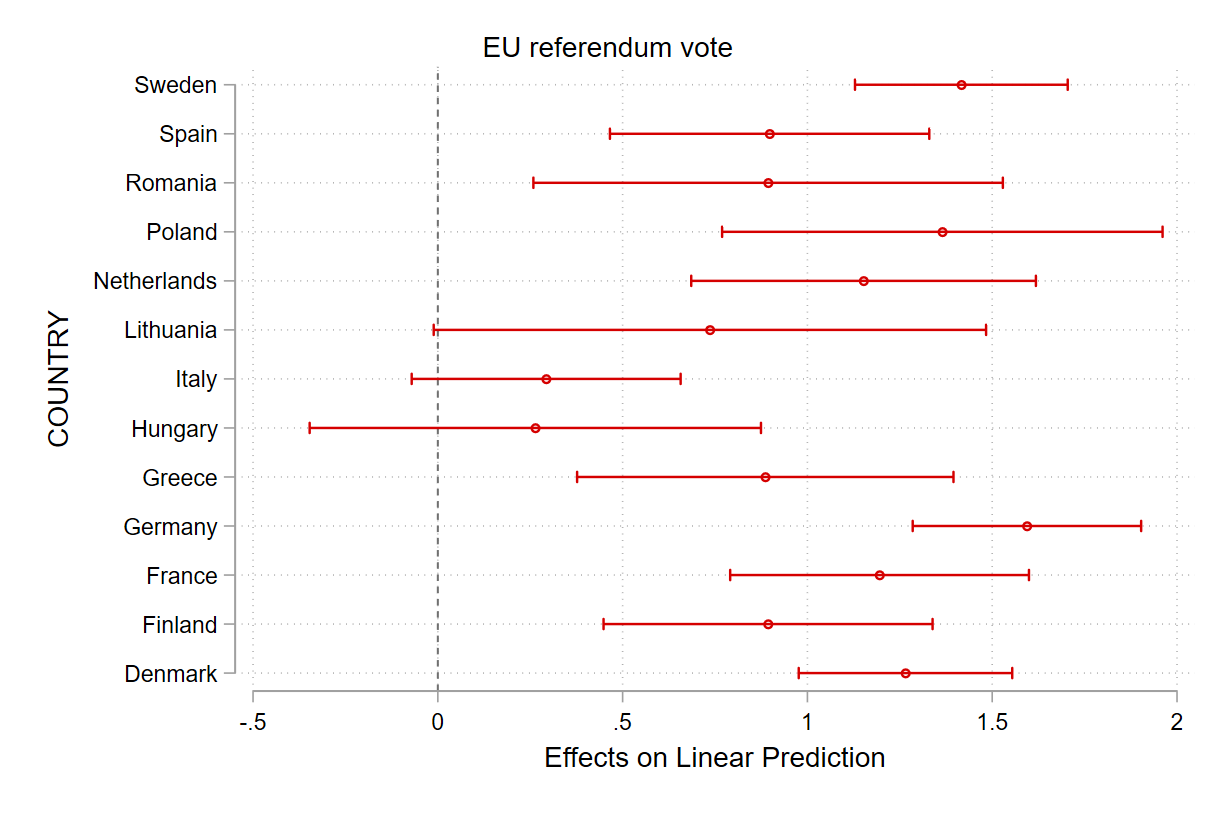

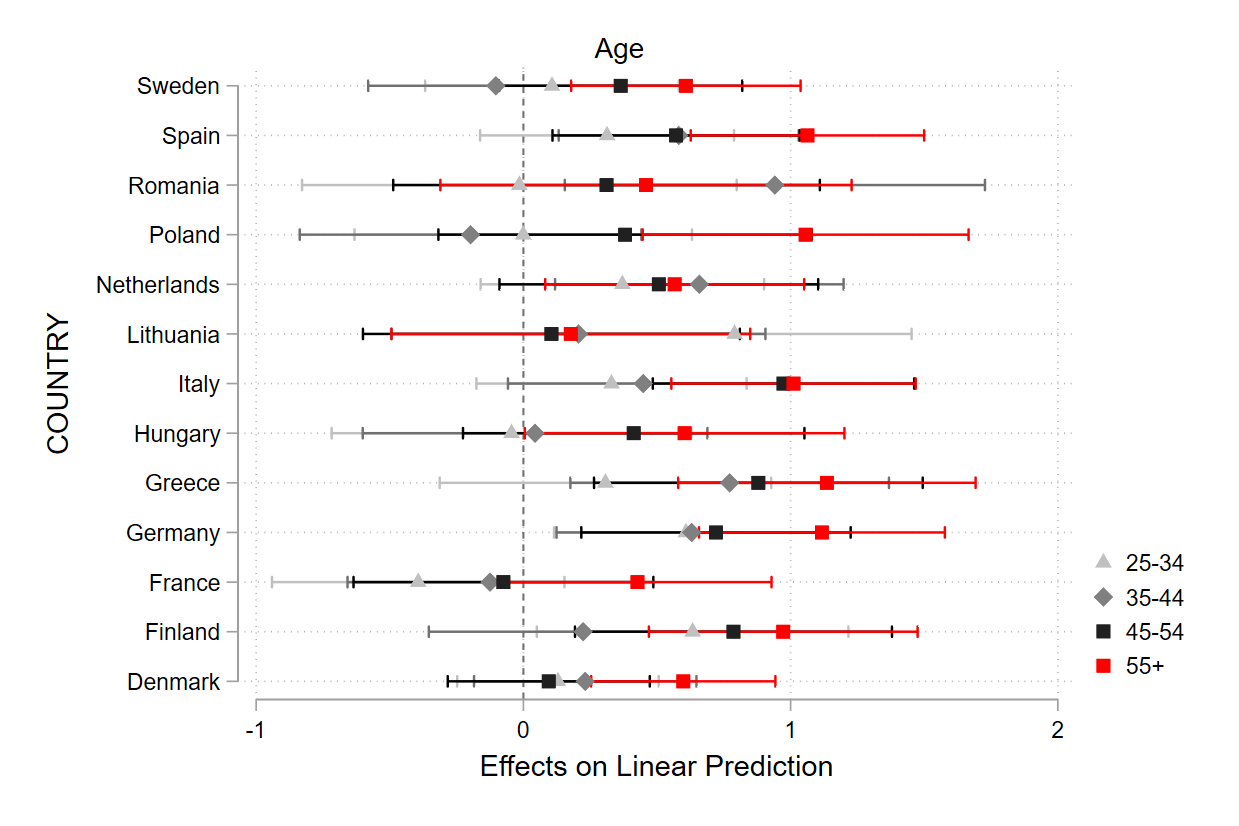

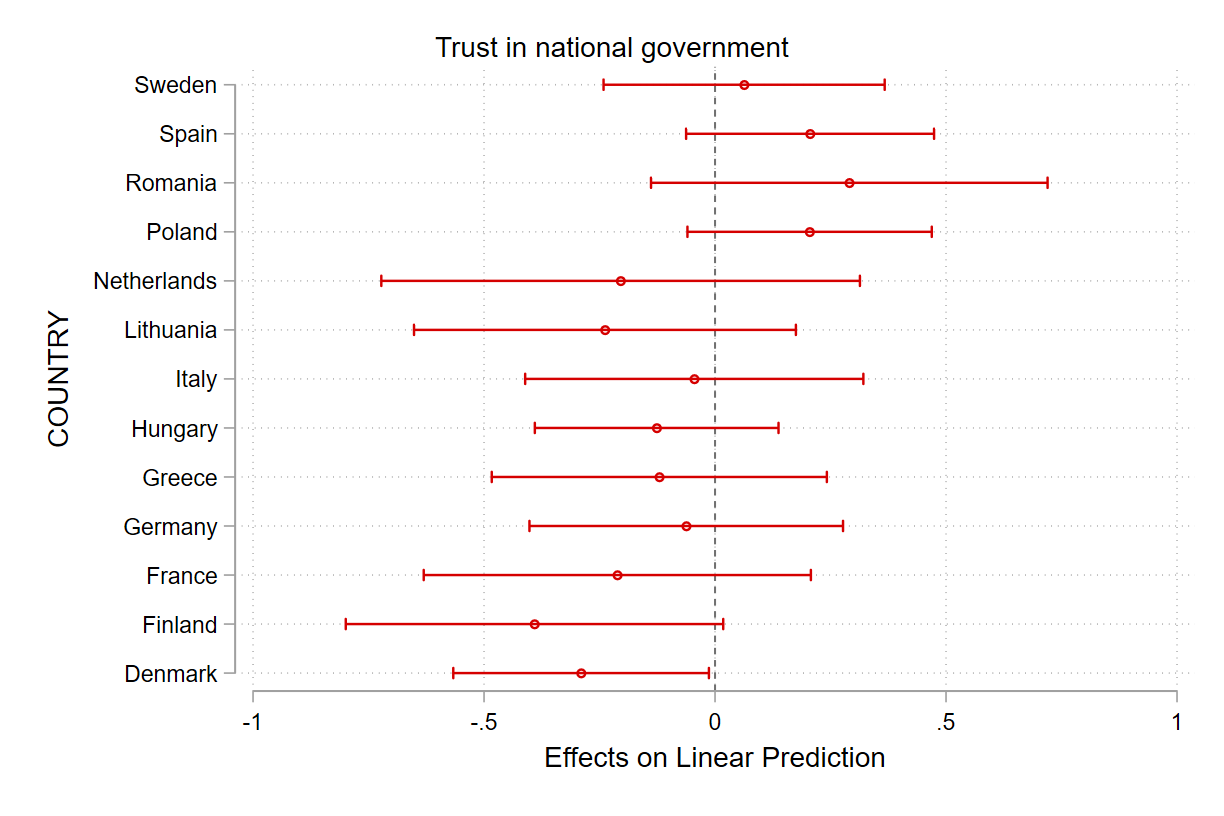

Supplement: Supplementary file 1 — Supplementary file1 (DOCX 963 kb) [file 41295_2023_345_MOESM1_ESM.docx]
